# The Impact of Vaccination Frequency on COVID-19 Public Health Outcomes: A Model-Based Analysis

**DOI:** 10.3390/vaccines13040368

**Published:** 2025-03-30

**Authors:** Lin Yuan, Madison Stoddard, Sharanya Sarkar, Debra van Egeren, Shruthi Mangalaganesh, Ryan P. Nolan, Michael S. Rogers, Greg Hather, Laura F. White, Arijit Chakravarty

**Affiliations:** 1 Fractal Therapeutics, Lexington, MA 02420, USA; lin.yuan@fractaltx.com (L.Y.); madison.stoddard@fractaltx.com (M.S.); 2 Department of Microbiology and Immunology, Dartmouth College, Hanover, NH 03755, USA; sharanya.sarkar.gr@dartmouth.edu; 3 Department of Oncology, School of Medicine, Stanford University, Stanford, CA 94305, USA; debra.vanegeren@gmail.com; 4 Faculty of Medicine, Nursing and Health Sciences, Monash University, Melbourne, VIC 3800, Australia; shruthi.mangalaganesh@gmail.com; 5 Halozyme Therapeutics, San Diego, CA 92130, USA; publications@halozyme.com; 6 Department of Surgery, Harvard Medical School, Boston, MA 02114, USA; michael.rogers@childrens.harvard.edu; 7 Vascular Biology Program, Boston Children’s Hospital, Boston, MA 02115, USA; 8 Sage Therapeutics, Cambridge, MA 02142, USA; ghather@gmail.com; 9 School of Public Health, Boston University, Boston, MA 02118, USA; lfwhite@bu.edu

**Keywords:** COVID-19, agent-based modeling, SARS-CoV-2, vaccination, scheduling, vaccine boosting

## Abstract

**Background:** While the rapid deployment of SARS-CoV-2 vaccines had a significant impact on the ongoing COVID-19 pandemic, rapid viral immune evasion and waning neutralizing antibody titers have degraded vaccine efficacy. Nevertheless, vaccine manufacturers and public health authorities have a number of options at their disposal to maximize the benefits of vaccination. In particular, the effect of booster schedules on vaccine performance bears further study. **Methods:** To better understand the effect of booster schedules on vaccine performance, we used an agent-based modeling framework and a population pharmacokinetic model to simulate the impact of boosting frequency on the durability of vaccine protection against infection and severe acute disease. **Results:** Our work suggests that repeated dosing at frequent intervals (three or more times a year) may offset the degradation of vaccine efficacy, preserving the utility of vaccines in managing the ongoing pandemic. **Conclusions:** Given the practical significance of potential improvements in vaccine utility, clinical research to better understand the effects of repeated vaccination would be highly impactful. These findings are particularly relevant as public health authorities worldwide have reduced the frequency of boosters to once a year or less.

## 1. Introduction

As the ongoing COVID-19 pandemic approaches its sixth year, the utility of vaccines in mitigating the death and disability burden of SARS-CoV-2 continues to evolve. Initial reports were consistent with strong vaccine protection against symptomatic disease, giving rise to the hope that vaccines could be used to achieve herd immunity to SARS-CoV-2 [1,2,3]. However, this promise was quickly undermined by rapid declines in vaccine efficacy against infection [4,5] driven by waning antibody titers [6,7,8,9] and viral immune evasion [9,10,11,12,13].

With herd immunity off the table, public health organizations pivoted to relying on vaccinations to manage the mortality burden of COVID-19, even as transmission continued. As vaccine efficacy against severe disease (VE_s_) was initially very high [1,2,3], this strategy contributed to a lowering of the infection fatality rate for SARS-CoV-2 [14]. Unfortunately, antibody waning and continued viral evolution have degraded VE_s_ [15,16,17,18], although it is partially restored with updated boosters [19,20,21] targeting newer variants.

Despite their limitations, the current crop of SARS-CoV-2 vaccines continues to form the centerpiece of public health strategies to manage the death and disability burden of COVID-19. At present, there are few nonpharmaceutical interventions (NPIs) mandated in any setting [22,23], while options for the treatment of serious disease are limited [24,25] and in some cases have been rendered obsolete by viral evolution [26,27,28].

On the bright side, immunological correlates of protection for SARS-CoV-2, particularly for infection, have been established, which is a boon to the rational optimization of vaccine performance. Neutralizing antibody (nAb) titers are a validated correlate of immune protection [29,30,31] for SARS-CoV-2. nAb titers normalized to mean convalescent titer (from the same study) have been shown to fit well to a nonlinear dose–response relationship that is predictive of reported vaccine protection across a range of different vaccines [32]. Two such dose–response curves exist, one linking nAb titers to protection against symptomatic infection and one linking nAb titers to protection against severe COVID-19 outcomes. These relationships have held up across a range of studies [33,34] and against newly emerging variants [21,35,36,37,38]. In these studies, nAb titers have been demonstrated to predict waning vaccine efficacy against infection (VE_i_) due to pharmacokinetic effects, as well as due to viral immune evasion. Waning nAb titers have also been demonstrated to be predictive of loss of VE_s_, although in this case, the 95% confidence intervals for the threshold of protection against severe disease are wide, indicating greater statistical uncertainty. It bears mentioning that the observed reductions in VE_s_ are inconsistent with the widely held perception [39,40,41] that the observed durability of T cell responses [42,43,44] would provide sustained vaccine protection against severe disease. (See Supplementary Section S1 for further discussion on the role of T cells in the vaccine and natural immune response to SARS-CoV-2.)

At present, the utility of vaccines in managing the mortality and morbidity burden of COVID-19 is limited. A recent CDC study showed that the vaccine effectiveness (a measure of VE_i_) of a bivalent mRNA COVID-19 booster received after two or more doses of monovalent vaccines ranged from 43% (for the 18–49 age group) to 22% (for the over-65 age group) [20]. When it comes to severe acute disease, VE_s_ for a newly boosted individual is now 56% [45], a steep decline from the originally reported VE_s_ (~100%) for COVID-19 mRNA vaccines [1,46]. A substantial portion of this drop in VE_s_ is likely to occur as a result of waning antibody titers and the viral evolutionary evasion of the immune response. (However, an increase in immunity due to infection among the unvaccinated also accounts for some of this apparent reduction in vaccine effectiveness [47].) Despite the observed losses in VE_s_ and VE_i_, public health organizations, including the CDC and WHO, have moved to an annual or less frequent boosting schedule for SARS-CoV-2 for the general population [48,49]. For the US population, this represents a sharp (about three-fold) reduction in recommended vaccine dosing frequency relative to the initial pace of boosters during the first 18 months following vaccine rollout. Even at this reduced frequency, uptake among adults is limited. For example, in the United States, only 22.5% of adults have received the updated booster released in winter 2023/2024 [50]. In many countries, access to boosters is limited to the elderly and/or immunocompromised [51,52,53], implying that the general population in those countries has very limited vaccine-derived protection. (We note that both the CDC and the WHO recommend twice-yearly vaccination for the highest risk groups [48,49].)

In addition to population-level waning in nAb titers, significant interindividual variation in the strength and durability of the nAb response also complicates the picture. In a prior study, we applied mixed-effects modeling to published SARS-CoV-2 nAb titers post-vaccination [54] and found a wide range of half-lives, with a 95% confidence interval ranging from 33 to 320 days. Lastly, as the pandemic has progressed, it has become clear that the delayed post-acute sequelae of COVID-19 (“long COVID”) also represent an important component of the morbidity burden of SARS-CoV-2 infections [55,56], with the potential for substantial impact on population-level health and economic outcomes [57,58,59]. The risk of long COVID upon infection has been shown to be reduced by up to around half following vaccination, in a number of studies and meta-analyses [56,60,61,62,63,64,65]. (However, considerable nuance exists in interpreting these results as long COVID is defined differently in each study, and the uninfected cohort is often not identified rigorously.)

Given our reliance on vaccines as a COVID-19 control measure and the observed decline in vaccine efficacy, creating a strong quantitative framework for understanding vaccine performance is helpful from a practical standpoint. At this juncture in the pandemic, it may be particularly helpful to ask two questions about SARS-CoV-2 vaccines: “are there ways to use the existing vaccines more effectively through optimization of boosting frequency?” and “what should vaccine makers focus on for the next generation of vaccines?” To answer these questions, we developed a longitudinal pharmacokinetic/pharmacodynamic (PK/PD) model of nAb kinetics and coupled it with an agent-based modeling framework. Our model combines population heterogeneity in the durability of the nAb response with the dose–response relationships linking nAb titers to protection from mild and severe disease. We have used this modeling framework to examine population heterogeneity in vaccine protection over time and in response to viral immune evasion. We formulate and test a potential strategy for improving the practical utility of existing vaccines by altering the dosing interval of the vaccines. We also extend our investigation to hypothetical vaccines with improved durability of response.

## 2. Materials and Methods

### 2.1. Agent-Based Simulation of Infection and Mortality Burden

#### 2.1.1. Model Overview

**Figure 1:**
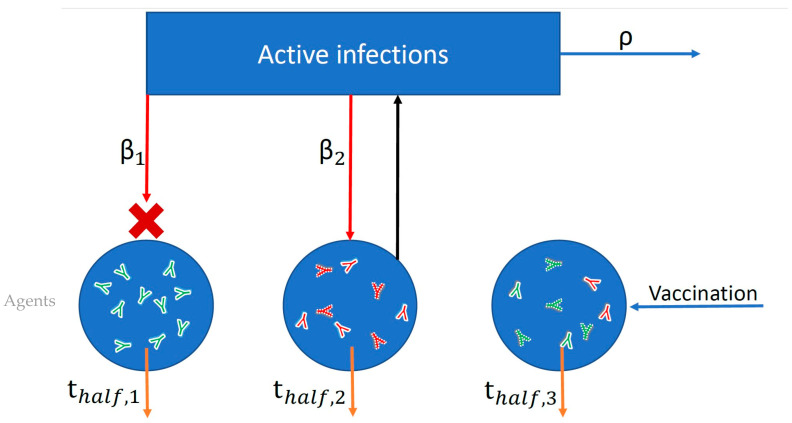
Schematic of simplified agent-based model used to simulate probabilities of infection and adverse outcomes based on nAb titers. Each blue circle represents an individual agent in the simulation. Within each agent *I*, neutralizing antibodies (nAbs) are generated as a result of vaccination (green antibodies) or infection (red antibodies), and these nAbs then decay over time according to each individual’s half-life (*t*_half,i_). Both infection and vaccination boost an individual’s nAb level by a fixed multiple. The blue rectangle represents the force of infection in the population (active infections multiplied by *R*_0_). Each individual agent is exposed to infection (red arrows) according to its contact rate (*β_i_*) and the force of infection. Exposure succeeds or fails to induce infection probabilistically according to the level of protection afforded by an individual’s combined infection and vaccine nAb titer. In the figure, infection failed (red X) for Agent 1 but succeeded for Agent 2. The number of active infections is tracked, increasing by one for each successful infection and decaying according to the recovery rate (*ρ*).

To determine the impact of boosting regimes on endemic SARS-CoV-2 infections and mortality among the vaccinated and the unvaccinated, we implemented a modification of an agent-based simulation developed in our prior work [57]. The schematic in Figure 1 shows the general structure of the model.

In an agent-based simulation, large numbers of autonomous agents are simulated, interacting with each other under a defined set of conditions. We simulated the number of interactions for each agent with other agents in the simulation based on random draws from a contact rate distribution. For each interaction, the likelihood that this corresponds to an exposure to SARS-CoV-2 is based on the force of infection (the product of the number of active infections and the intrinsic reproductive number *R_0_* divided by the duration of infection). Once exposed, each agent has a probability of infection proportional to their level of immunity at that time (based on a simulation of that agent’s nAb titers). These simulated nAb titers are based on a pharmacokinetic mixed-effects model fit to experimental data for nAb titers over time. A successful infection boosts the individual’s nAb titers by a fixed multiple and increases the total count of active infections. Vaccination occurs at a fixed frequency and boosts vaccinated individuals’ vaccine nAb titers. We assumed that 50% of the population receives boosters at staggered intervals, which vary from two months to one year. The booster uptake is roughly consistent with the US first booster uptake in 2021, but considerably higher than the current rate of booster uptake in the US (23% [50]) (The implications of a higher booster rate are discussed in the limitations section of Section 4).

We used this simulation to model SARS-CoV-2 spread under endemic conditions to evaluate the relationship between booster frequency and the range of outcomes in the boosted population. The model simulates outcomes for 100,000 individuals over 10 years.

#### 2.1.2. Population Mixed-Effects Model Fit for Neutralization Potency over Time After Infection

To determine the kinetics of serum nAb titers after SARS-CoV-2 infection, we applied a two-stage model structure to the nAb titer dataset published by Wang et al. [66]. The two stages of the model are the production phase, in which antibody titers are both produced and eliminated, and the memory phase, where antibodies are only eliminated. The transition from the production phase to the elimination phase occurs at time *T*. We compared first-order and zero-order production models, and the zero-order production model was selected for neutralization potency based on lower AIC and good parameter estimation with low standard errors. The following equations describe the zero-order and first-order models:

Zero-order:

$$\frac{dA}{dt}=k_{p}-k_{el}A\;\left(t<T\right)\;\frac{dA}{dt}=-k_{el}A\;\left(t\;\geq \;T\right)$$

First-order:

$$\frac{dA}{dt}=k_{p}A-k_{el}A\;\left(t<T\right)\;\frac{dA}{dt}=-k_{el}A\;\left(t\;\geq \;T\right)$$

where *A* is the antibody titer, *k_p_* is the rate of antibody production, *k_el_* is the rate of antibody elimination, and *T* is the duration of antibody production. Model parameter values are shown in Table 1. The dataset contains nAb titers for 30 patients who recovered from COVID-19 and were discharged from the Yongchuan Hospital of Chongqing Medical University. Age and gender were statistically analyzed as covariates in Monolix (MonolixSuite2024R1, Lixoft SA, Paris, France). A goodness-of-fit analysis confirmed model specification and fit to the dataset (Supplementary Figure S2).

We modeled the distribution of nAb half-lives after vaccination similarly, using parameters derived from a previous mixed-effects model fit that we conducted on a nAb titer dataset derived from adults vaccinated with the Moderna mRNA-1273 vaccine [54].

The distribution of antibody elimination rates from the mixed-effects model fits was converted to distributions of nAb half-lives according to the relation *t*_1/2_ = ln(2)/*k_el_* (Supplementary Figure S3).

#### 2.1.3. Agent-Based Simulation of SARS-CoV-2 Dynamics

Under endemic conditions, individuals in the population will become reinfected with SARS-CoV-2 as their neutralizing antibody titers wane. To simulate the impact of heterogeneous natural immunity on long-term SARS-CoV-2 infection dynamics and fatality rates, we developed a simplified agent-based epidemiological model that accounts for interindividual heterogeneity in the rate of antibody waning and in exposure risk (contact behavior). This model tracks the nAb titers of simulated individuals with fixed contact rates, neutralizing antibody decay rates, ages, and vaccination statuses. The individuals’ contact rates are parameterized by a random draw of the contact distribution [67], while individual ages are set based on a random draw of the age distribution of the United States [68]. Each individual’s half-lives for vaccine- and infection-derived nAbs are randomly drawn from the corresponding distributions from the population PK model fits. Contact rates are treated as relative rather than absolute—the model’s intrinsic reproductive number (*R*_0_) multiplied by the normalized individual contact rate determines the individual’s absolute rate of exposure.

For each individual, the cumulative number of infections and neutralizing antibody titer are tracked over time. The neutralizing antibody titer of each individual wanes according to the individual’s decay rate and the rate of immune evasion through viral evolution. For each individual, the titer is updated at each timestep as follows:

$$titer\left(t+dt\right)=titer\left(t\right)*e^{-\frac{0.693*dt}{\left(t_{\frac{1}{2},waning}+t_{\frac{1}{2},\;evasion}\right)\;}}$$

where *t*_1/2,_*_waning_* is the individual’s neutralizing antibody half-life and *t*_1/2,_*_evasion_* is the half-life of nAb potency due to immune evasion.

Additionally, neutralizing antibody titers are boosted by a fixed multiple when the individual is successfully infected after an exposure or after vaccination. All individuals in the population are exposed at a rate proportional to their contact rate. This is implemented as a random draw on the simulated population, with each individual’s likelihood of being drawn proportional to their contact rate. Upon exposure, an individual’s risk of infection is calculated based on their neutralizing antibody titer. The risk of infection upon exposure is

$$p(infection\;|\;exposure)=1-\frac{titer^{h}}{titer^{h}+E{C_{50,\;infection}}^{h}}$$

where *titer* is the individual’s neutralizing antibody titer normalized to peak convalescent level, *h* is the Hill coefficient, and *EC*_50,*infection*_ is the neutralizing antibody titer required for 50% protection from infection. *EC*_50,*infection*_ was assumed to be 35% of the peak convalescent plasma level because we found this to be approximately consistent with clinical data suggesting median protection from reinfection is 83% over 5 months after infection [69]. The exposure results in successful infection if a random value between 0 and 1 is less than *p (infection|exposure).* Then, the individual’s antibody titer is boosted by a fixed multiple [32].

We assumed that all infections are of the same duration, and the number of active infections is tracked using a counter. The counter is increased by one for each successful infection, while recovery events decrease the counter by one. Each active infection has a 10% likelihood of recovery per day, based on an average 10-day infection duration [70]. The rate of exposures is determined by the force of infection (the expected number of secondary infections in the absence of pre-existing immunity), given by the number of active infections multiplied by *R*_0_. This determines how many exposures occur at each timestep, with exposures either succeeding or failing to induce an infection as described previously. As such, we do not account for overdispersion (variability in infectivity of individuals), and we assume that the population is well mixed (any individual can infect any other individual, with no network effects).

Vaccination is modeled similarly to infection, with neutralizing antibody titers increased by a fixed multiple upon vaccination. To account for differences in persistence of post-vaccination and post-infection nAbs, the model separately tracks nAb titers induced by vaccination and infection. Each member of the simulated population is randomly designated as “vaccinated” or “unvaccinated” in a proportion consistent with the specified prevalence of vaccination in the population. Upon vaccination or infection, an individual’s nAb titers are increased by a fixed multiple (revaccination 10-fold [71] and reinfection 14.4-fold [72]). Conservatively, we assumed that any antibodies generated through revaccination (by boosting of pre-existing vaccine or infection-induced antibodies) wane at the vaccine nAb decay rate. All vaccinated individuals are boosted (revaccinated) at a fixed interval, which is tracked by an individual counter for the simulation days since last vaccination. These intervals are staggered among the individuals in the simulated population. Once an individual’s revaccination counter reaches their revaccination interval, the individual’s antibody titers are multiplied by the vaccination multiple, and the counter is reset to zero.

In addition to tracking each simulated individual’s number of infections, we calculated their cumulative risk of fatal COVID-19. For each individual, we calculated an immunologically naïve infection fatality rate (IFR) based on their age according to a published formula [73]. We adjusted the population average IFR according to this formula to reflect estimates for omicron’s IFR (0.21%) [74].

Upon infection, an individual’s risk of fatal disease is calculated based on their normalized neutralizing antibody titer:

$$p(death\;|\;infection)=\frac{p(death\;|\;exposure)}{p(infection\;|\;exposure)}$$

when

$$p(death\;|\;exposure)=\left(1-\frac{titer^{h}}{titer^{h}+E{C_{50,\;death}}^{h}}\right)*naive\;IFR$$

In this case, *EC*_50,*death*_ is the neutralizing antibody titer required for 50% protection from death; in this analysis, we assumed *EC*_50,*death*_ is equal to the *EC*_50_ for protection from severe disease parameterized by Khoury et al. (3% of CP titer) [32]. (Although this number was assessed prior to the Omicron variant, subsequent analyses show that the threshold has not varied by variant.) Each individual’s cumulative probability of survival is tracked, with the probability starting at 1 and being multiplied by the risk of death upon each infection. The population-level death count is increased by the expected value of death imposed by each infection (that is, the individual’s risk of death for that infection).

The model is designed to capture steady-state (endemic) SARS-CoV-2 dynamics. Before each simulation, the model is run for 1000 days to reach equilibrium. As a result, the initial values of simulated individuals’ nAb titers are arbitrary. To demonstrate both stochastic year-to-year variation in individual outcomes and long-term average risks, we ran simulations over 1 year and over 10 years after equilibrium conditions were reached.

Thus, this model accounts for interindividual heterogeneity in nAb waning rates after vaccination [54] and infection [57], a steady rate of nAb potency loss due to immune evasion, variability in contact behavior between individuals, and variation in severe disease susceptibility due to age.

For the simulations in the main text, we assumed that 50% of the population is vaccinated, which is in rough agreement with the fraction of the eligible population that has received a first booster in the US [75]. This number, while higher than the current booster uptake rate [50], was selected as an achievable uptake rate. Model parameters for the agent-based simulation are summarized in Table 2.

### 2.2. Susceptible–Infectious–Recovered–Susceptible (SIRS) Model of Strain Invasion

We also performed a retrospective analysis to determine whether a delta-like wave could have been avoided by administering boosters to all US adults under conditions similar to the summer of 2021. For this purpose, we adapted the two-strain SIRS model with vaccines we implemented in our prior work predicting variant-driven waves after vaccine rollout [75]. We assumed that the alpha variant dominated prior to delta emergence. We assessed multiple scenarios for vaccine efficacy against infection (Vei) based on clinical data for recent two-dose primary series, distant (>90 days) primary series, and a recent first booster. We assessed best-case vaccination scenarios in which all US adults were vaccinated and realistic scenarios in which 48% of the population was vaccinated (Table 3).

## 3. Results

### 3.1. Boosting Frequency Determines Vaccine Efficacy Throughout the Population

**Figure 2:**
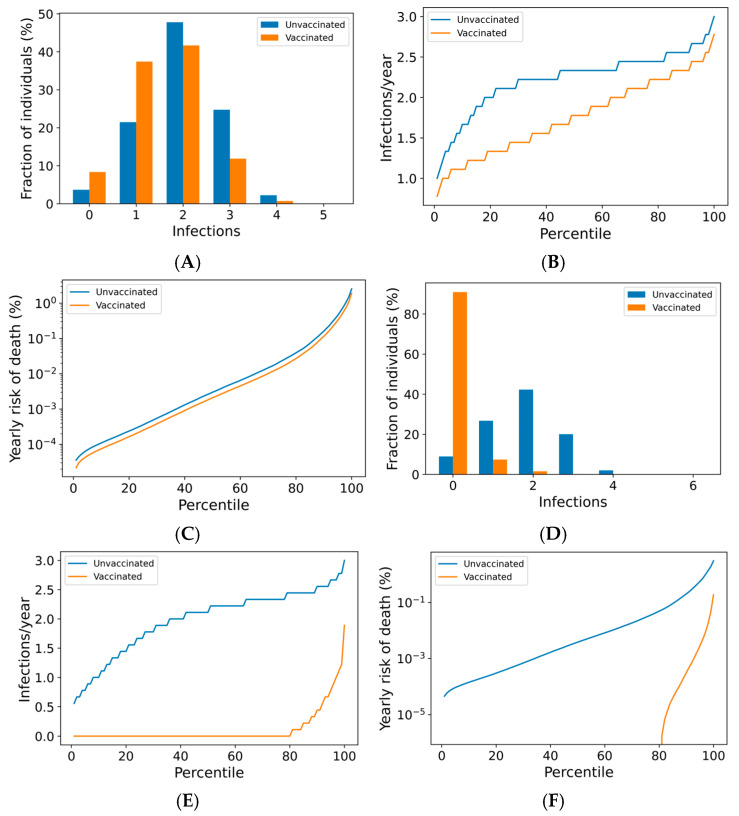
Frequency of boosting determines frequency of infection and risk of COVID-19 death among the vaccinated. For a once-yearly booster frequency, (**A**) the distribution of infection counts in a single year, (**B**) the distribution of individual infection frequencies over a 10-year simulation, and (**C**) interindividual heterogeneity in yearly risk of COVID-19 death. For a four-times yearly booster frequency, (**D**) the distribution of infection counts in a single year, (**E**) the distribution of individual infection frequencies over a 10-year simulation, and (**F**) interindividual heterogeneity in yearly risk of COVID-19 death. On a short-term basis (**A**,**D**), variation in infection risk is driven by interindividual heterogeneity in biology and behavior as well as stochasticity. In the long term (**B**,**C**,**E**,**F**), interindividual heterogeneity dominates stochasticity in driving individual infection frequency and severe disease risk.

We now use the agent-based simulation in Section 2.1.3 to examine the impact of boosting regimes on endemic SARS-CoV-2 infections and mortality among the vaccinated and the unvaccinated. The distributions of outcomes in both unvaccinated and vaccinated subpopulations are shown in Figure 2. Once-annual boosting provides some benefit for reducing the frequency of infection and risk of death among the vaccinated (Figure 2A–C). In a given year, over 90% of vaccinated individuals are expected to experience SARS-CoV-2 infections. On average, the median boosted individual experiences approximately 1.5 infections per year, while the unvaccinated median individual is infected slightly more than twice yearly. The risk of death for the median vaccinated individual is predicted to be approximately half that of the median unvaccinated individual.

The outcomes for the vaccinated population improve as boosting frequency increases (Figure 2D–F, Supplementary Figures S7 and S8). The vast majority of individuals receiving four boosters per year are not infected in any given year (Figure 2D)—over 80% of these individuals are never infected within a 10-year simulation period (Figure 2E). The annual risk of death greater than 0.1% is extremely rare in the quarterly boosted population (<2%), while 15% of the unvaccinated population experiences a risk of this magnitude (Figure 2F).

Vaccine impact on the risk of infection for a vaccinated individual can be broken down into first-order (vaccination protects the vaccinee upon exposure) and second-order (vaccination reduces transmission in the population, which protects everyone) effects. In Supplementary Figure S10, we separated first-order from second-order effects by considering the outcomes of a negligibly small subpopulation (5%) of vaccinated individuals. Our results suggest that the benefits to individuals from a denser booster schedule are largely unchanged, even under conditions of low vaccine compliance.

### 3.2. Breakthrough Infections Under Frequent Boosting Schedules Are Driven by Poor nAb Kinetics

**Figure 3:**
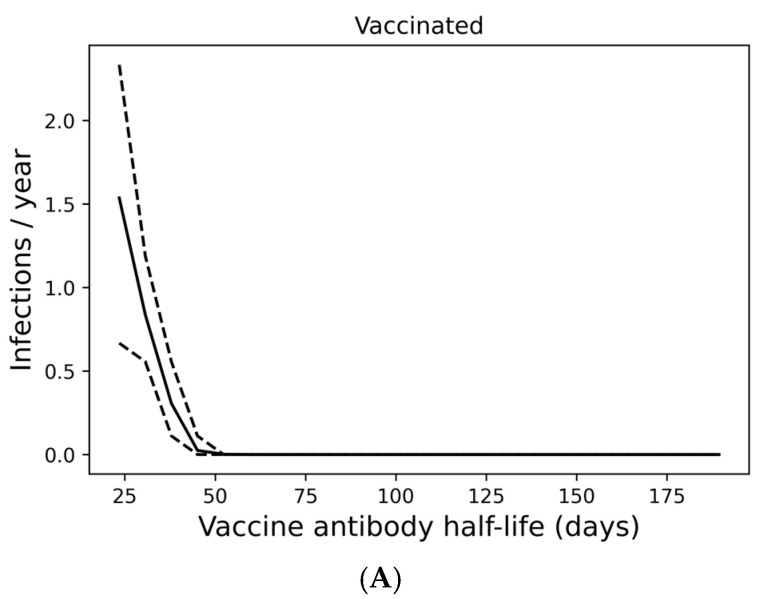

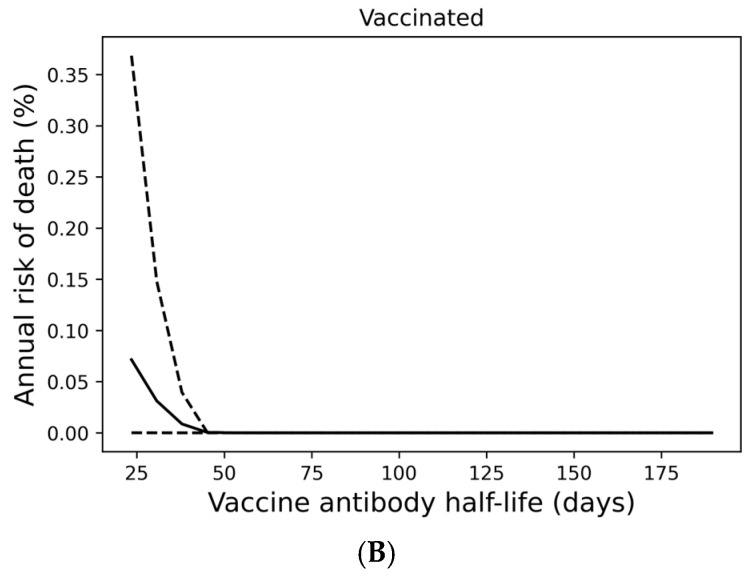
Infection despite four times yearly vaccination is strongly predicted by short vaccine antibody half-lives. (**A**) Average infection frequency and (**B**) average yearly risk of death over a 10-year simulation. Dashed lines represent the 90% population interval.

Although most individuals avoid SARS-CoV-2 infection entirely under a quarterly boosting schedule, 17% of boosted individuals are expected to become infected at any frequency over a 10-year period, with 10% experiencing infection at least every other year (Figure 2E). In Figure 3A, we demonstrate that infection despite quarterly revaccination is strongly predicted by short vaccine nAb half-life. While many individuals with vaccine nAb half-lives less than 50 days are infected, no one with a half-life greater than 50 days is infected in a 10-year simulation. These infections impose a significant risk of death, especially for those with the least persistent vaccine antibodies (Figure 3B). However, the identification of these individuals and the application of more frequent boosting could improve outcomes in this population. We found that six vaccinations per year could suppress infection in those with the shortest vaccine nAb half-lives observed in the immunocompetent population (Supplementary Figure S4).

### 3.3. High Compliance with Frequent Boosting Could Suppress Omicron Spread

**Figure 4:**
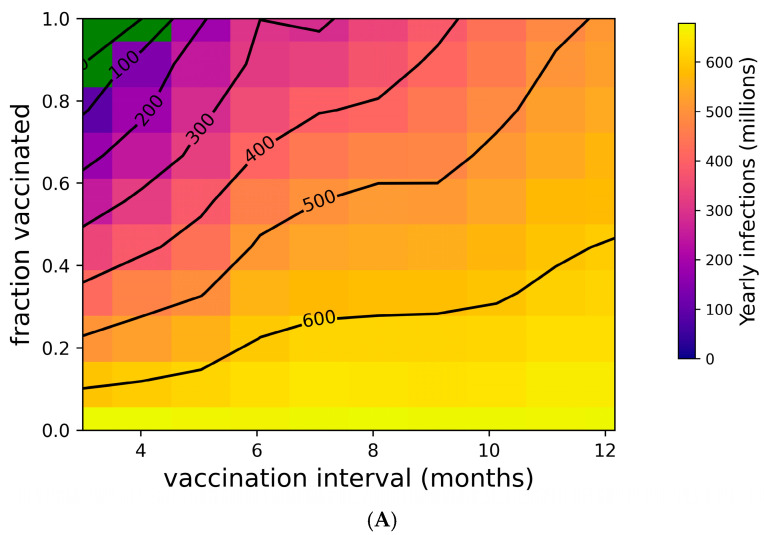

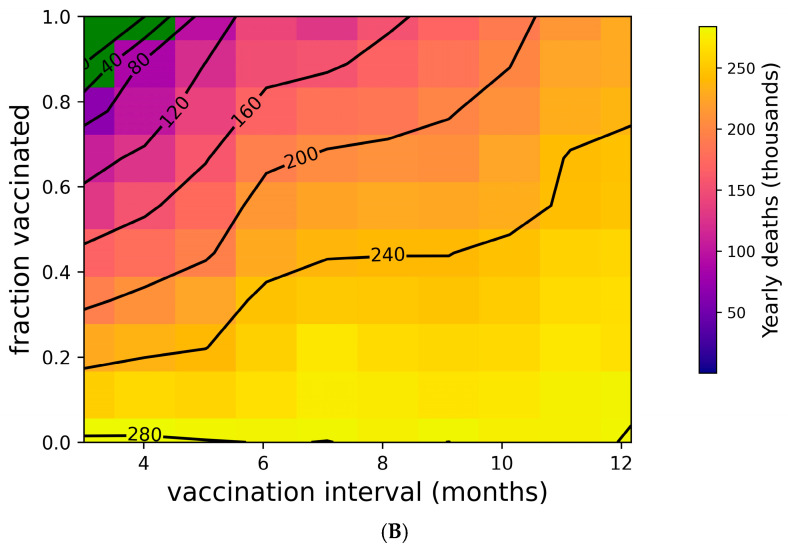
Suppression of SARS-CoV-2 can be achieved with sufficiently high vaccination rates. (**A**) Yearly US SARS-CoV-2 infections and (**B**) yearly US COVID-19 deaths under a variety of vaccination frequency and compliance scenarios. Green region represents complete suppression (zero infections at steady state).

Figure 2 addresses the distribution of individual outcomes under various boosting regimes, while Figure 4 shows the population-level impact of boosting frequency and compliance. Frequent boosting coupled with high compliance is predicted to substantially reduce the impact of COVID-19 at the population level. Despite the high transmissibility of omicron (R_0_ = 8.2 [76,77,92]), the complete suppression of spread is possible with a high degree of compliance and frequent boosting (i.e., approximately 90% compliance with boosters every three months, or perfect compliance with boosters every four months) (Figure 4A). The vaccine’s impact on yearly death tolls is even more dramatic (Figure 4B). In the absence of the complete suppression of SARS-CoV-2 spread and increased vaccination coverage and frequency can reduce yearly death tolls. For example, if 50% of the population is vaccinated, an increase in vaccination frequency from once yearly to twice yearly could avert approximately 40,000 US COVID-19 deaths. Increasing vaccination coverage to 90% could prevent an additional 50,000 US COVID-19 deaths. As shown in Supplementary Figure S6, vaccines with superior kinetics could further reduce infections and deaths in addition to widening the space for complete disease suppression.

### 3.4. Improved Vaccine Kinetics Improves Booster Regime Efficacy

The Moderna mRNA COVID-19 vaccine induces less durable nAbs relative to post-infection immunity [57,93]. We surmise that in a future vaccine development program, the target product profile for such a vaccine could be based on ensuring that the distribution of nAb half-lives is improved to match or exceed post-infection immunity.

**Figure 5:**
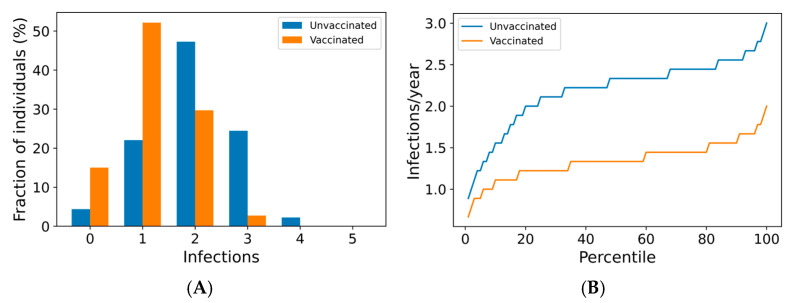

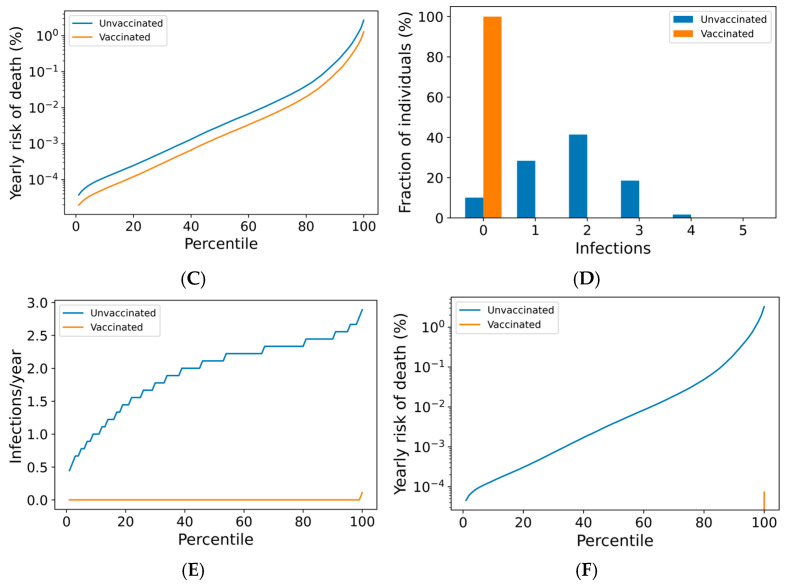
Three yearly boosters may prevent infection in nearly all vaccinees for a vaccine with nAb kinetics similar to post-infection. For a once-yearly booster frequency, (**A**) the distribution of infection counts in a single year, (**B**) the distribution of individual infection frequencies over a 10-year simulation, and (**C)** interindividual heterogeneity in yearly risk of COVID-19 death. For a three-times yearly booster frequency, (**D**) the distribution of infection counts in a single year, (**E**) the distribution of individual infection frequencies over a 10-year simulation, and (**F**) interindividual heterogeneity in yearly risk of COVID-19 death. On a short-term basis (**A**,**D**), variation in infection risk is driven by interindividual heterogeneity in biology and behavior as well as stochasticity. In the long term (**B**,**C**,**E**,**F**), interindividual heterogeneity dominates stochasticity in driving individual infection frequency and severe disease risk.

To evaluate the impact of such improvements, we reimplemented our analysis under the assumption that post-vaccination nAbs have the same kinetics as post-infection nAbs (Figure 5, Supplementary Figure S5). In other work, we found the population median half-life of post-infection nAbs to be 109 days [57]. In this case, the impact of yearly boosters is improved, with lower infection and death rates overall and less variation in outcomes (Figure 5A–C). With a hypothetical vaccine possessing these characteristics, only 3 yearly boosters would be required to achieve near-complete protection from infection in the vaccinated population.

### 3.5. Boosting Could Have Likely Averted the Delta Wave of Summer 2021

**Figure 6:**
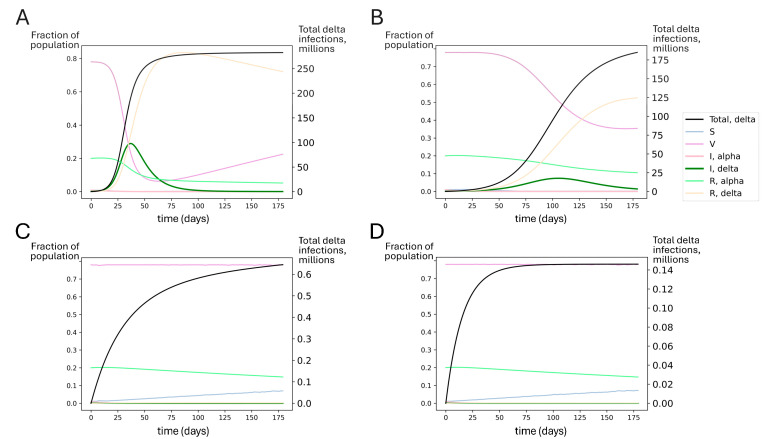
Simulated outcomes for booster rollout to all US adults under conditions similar to delta’s emergence. Each compartment in the SIRS model is represented by the fraction of individuals in the population in that compartment over time (left axis). S represents susceptible, V represents vaccinated, I represents infected with alpha or delta, and R represents recovered from alpha or delta. The total number of delta infections is tracked (black line, right axis). Regardless of the extent of the nonpharmaceutical mitigation of SARS-CoV-2 spread, booster vaccinations for all adults could have significantly reduced delta spread compared to estimated vaccine effectiveness at the time (waned primary series). Model-predicted delta variant outbreak dynamics assuming (**A**) no booster rollout and no mitigations; (**B**) no booster rollout and mitigations reducing transmission by 50%; (**C**) booster rollout to all US adults with no additional mitigations; and (**D**) booster rollout to all US adults with additional mitigations reducing transmission by 50%. The SIRS model suggests that perfect adult compliance with a booster campaign before delta became dominant could have averted the delta wave.

Next, we used an SIRS model to explore theoretical scenarios similar to the emergence of the delta variant in summer 2021 in the United States. In the early summer, alpha was dominant but in decline, and 48% of the US population was fully vaccinated (see Table 3). As summer progressed, the delta variant established itself in the US [94], leading to a surge in infections. In Figure 6A, we show that unmitigated spread of delta without a booster campaign could have impacted most of the US population (fortunately, infections are estimated to have been limited to approximately 15% of the population [95,96,97]). Modest (50%) mitigations could have reduced the overall toll, but a large infection count would still be expected (Figure 6B).

However, these outcomes were not inevitable. Even in the absence of NPIs, if all adults had been boosted recently before delta introduction, this could have significantly blunted the impact of the delta surge. Achieving boosting in all US adults could have dramatically improved outcomes, suppressing delta spread to very low levels (Figure 6C,D). In Figure S6, we show that boosting only the 48% of Americans who had received the primary series by summer 2021 could have significantly reduced the spread of the delta variant.

## 4. Discussion

As the SARS-CoV-2 pandemic continues to pose a public health threat, there remains a significant degree of interest in exploring improved vaccine designs and formulations through the discovery and development of next-generation vaccines. In contrast, the work presented here explores opportunities for improving the performance of the current generation of SARS-CoV-2 vaccines in mitigating the morbidity and mortality burden of COVID-19. Our findings suggest that vaccine performance may be improved through booster schedule optimization and improved nAb kinetics, providing insights that may be leveraged in the design of further clinical trials. Crucially, our work suggests that boosting three or more times a year may preserve VE_s_ and potentially restore VE_i_. Conversely, our findings suggest that a booster dose frequency of once a year is unlikely to provide a significant population-level benefit from vaccines under endemic SARS-CoV-2 conditions, though individuals may experience transient protection in the post-booster interval. Given the low frequency of vaccine uptake in the US adult population at this point, it is therefore likely that vaccines at their current schedule and uptake are providing only modest population-level (or individual) benefit against infection.

Improving vaccine protection against infection has particular significance at this stage of the pandemic, as a significant proportion of the severe outcomes of COVID-19 are now linked to the post-acute sequelae of infection. As long COVID risk is proportional to the number of infections [98], one can infer that a reduction in infection risk would have a significant impact on reducing the long-term harm associated with COVID-19 for individuals and the population.

In this work, we used population PK/PD modeling coupled with an agent-based simulation to interrogate the impact of booster scheduling on vaccine protection against infection and severe disease. As such, our recommendations provide practical guidance for further vaccine development studies while highlighting the limitations of the current understanding of the immune response to repeated vaccine dosing. Modeling studies have had substantial predictive value during the course of this pandemic. Our team has used model-based approaches to predict the rapid pace of evolutionary immune evasion [99], the inability of vaccines to enable a return to pre-pandemic conditions [100], the tendency of noncompliance with mitigation measures to incentivize further noncompliance [101], and the rapid variant-driven rebound observed upon premature relaxation of mitigation measures [75]. In each of these cases, our predictions were made months in advance [75,100,101,102].

That said, this work has limitations that provide important context for interpreting our findings. First, the extent to which nAbs accumulate upon repeated vaccine dosing is not fully characterized. Some reports have suggested that these antibodies continue to accumulate upon repeated boosting [103,104,105], while others have suggested a cap or maximum level of neutralizing antibodies [106] or the attenuation of response [107]. A recent study conducted by vaccinating Balb/c mice with repeated, closely spaced doses of recombinant RBD spike protein reported immune tolerance [108]. The study dosed mice with a vaccine every other day, a far denser schedule than what was used in humans, with no translational rationale for this altered schedule. The adjuvant used was also different from the clinical studies, and no rationale was provided for this. It is known that adjuvants play a substantial role in tolerogenicity in humans, and mouse immunology studies are known to be poorly predictive in a human context [109]. Additionally, tolerogenic effects in the study were only observed after the fifth dose, which used a different adjuvant than previous doses, raising the possibility that the change in adjuvant contributed to the change in response [108].

The degree to which homologous boosting with outdated vaccines can raise titers against novel variants is not fully known, nor is the extent to which future nAb responses are shaped by previous exposure (“immune imprinting” [110]). Despite these concerns, there are reasons for optimism. The bivalent wild-type/BA.5 booster increases neutralizing titers 1.3-fold compared to the monovalent wild-type booster [111], and it increases breadth against emerging immune-evasive variants [112]. Although this model assumes an individual’s nAb vaccine and post-infection nAb half-lives are fixed, emerging data suggest that nAb half-lives may be increased by repeat exposure [113,114,115,116]. Additionally, multiple studies suggest that repeat vaccination improves the breadth of the nAb response against novel variants [114] and antibody avidity [117].

The small population size of the underlying dataset reflects another limitation: the antibody data used to fit the PK model were derived from a Moderna Phase 1 trial enrolling 34 participants, with immunocompromised status being an exclusion criterion for the trial [6,54]. In the model, vaccination status was assumed to be age-independent, and long COVID outcomes were not considered. The contact distribution used for the agent-based modeling forms another limitation of the work, as it impacts the frequency of infection. In the context of this work, the relative reduction in infection risk upon increasing booster frequency, particularly the finding that denser booster dose frequencies may restore VEi, is not expected to be impacted. Finally, the impact of vaccine side effects or toxicity upon repeated dosing is not considered explicitly, as there is no dataset to define this. It is possible that vaccine toxicity could constrain the frequency of boosting. (We discuss this limitation further below.)

As a further limitation, we have not considered the role of compartments of the immune system beyond nAbs in the vaccine response. Several lines of evidence support this choice. The existing data provide strong support for nAbs as a validated correlate of immune protection [4,21,29,30,31,32,33,34,35,36,37,38,118,119] (see Supplementary Material S1, Footnote H). Consistent with this, lower nAb levels can lead to worse outcomes for SARS-CoV-2 infection [120,121,122,123,124,125,126,127,128,129,130,131,132,133,134,135,136,137,138,139,140,141] (Supplementary Material S1, Footnote I).

On the other hand, a growing body of research suggests a limited or nonexistent role for T cells in conferring protection against SARS-CoV-2 infection or severe disease [39,40,41,142,143,144,145,146,147,148,149,150,151,152]. Although T cell response is persistent and durable after infection and vaccination [42,43,44,136,137,138,139,140,141,153,154,155,156,157,158] (Supplementary Material S1, Footnote A), vaccine efficacy against severe disease (VEs) wanes rapidly and is vulnerable to immune evasion [1,4,8,16,17,18,20,21,45,46,87,159,160,161,162,163,164] (Supplementary Material S1, Footnote B). Further evidence of this disconnect between T cell activity and COVID-19 outcomes is apparent in COVID-19 patients who used the T cell blocking drug Abatacept, which has not been shown to impact disease outcome [165,166,167,168,169,170] (Supplementary Material S1, Footnote F). While the restoration of nAb levels in B cell-depleted patients can improve COVID-19 outcomes, boosting T cell function does not [88,131,171,172,173,174,175,176,177] (Supplementary Material S1, Footnote J).

Furthermore, although SARS-CoV-2-specific CD8+ and CD4+ T cells are elevated following infection, total T cell counts decrease during infection with SARS-CoV-2, because T cells are directly infected by SARS-CoV-2, triggering apoptosis [15,147,171,178,179,180,181,182,183,184,185,186,187,188,189,190,191,192,193,194,195,196,197,198] (Footnotes C, D). Nevertheless, the loss of T cells does not correlate with worse outcomes for COVID-19 disease progression [120,124,199,200,201,202,203,204,205,206,207,208,209,210] (Supplementary Material S1, Footnote E). Reports of T cell-mediated protection have suffered from poor interpretability due to confounding with nAb titers [169,200,211,212] (Supplementary Material S1, Footnote G). Based on these multiple lines of evidence, we assumed that the primary source of protection for COVID-19 is humoral and not cellular immunity [99,101,213,214,215,216]. (For a more in-depth discussion, see Supplementary Material S1).

To the extent that neutralizing antibodies are the primary correlates of immune protection against SARS-CoV-2, our work makes several crucial points for public health strategy. First, recommendations for boosting should be driven by science, not based on perceptions of what the public will find acceptable. Current public health messaging is ambiguous around the necessity of booster doses. For example, in the US, public health figures and the administration have expressed a preference for a once-a-year booster focused on the medically vulnerable [48,49,217], and even this has been met with skepticism in some quarters [218,219]. Our work shows that such a strategy would come at a significant human cost, causing VE_s_ to plummet. This messaging has been accompanied by a decline in vaccine uptake, with the monthly averages for US adult vaccines administered having declined ten-fold since the peak in the spring of 2021. As of December 2024, only 20% of US adults have received the most recent booster [50]. One poll, run by the Kaiser Family Foundation, found that 62% of adults are either unvaccinated or are not planning to take the booster at this point. Among people who had already received the original vaccine series but did not intend to receive a booster, the most common reasons for not seeking an additional booster were that they did not believe they needed one (44%) or they did not believe the benefits were worth it (37%) [220]. These numbers are striking given that among individuals who had received the third booster at least four months prior, a fourth booster has been demonstrated to halve infection risk [221] and reduce the risk of severe disease by up to 3.5-fold [222].

It is thus imperative that boosting frequency recommendations be set by public health authorities on a data-driven basis. Clinical studies exploring tolerability for dense booster schedules represent an area of shared interest for public health agencies and vaccine manufacturers. Such clinical studies are also of critical importance in understanding the potential for improved protection with denser booster schedules for older and vulnerable subpopulations. Our work points out the importance of dose schedule on potential vaccine performance—it is our recommendation that health authorities should work with vaccine regulators and manufacturers to more closely align the experimental clinical data that drive vaccine schedule recommendations.

If the goal is to implement an “individual public health” strategy, where each person is required to make their own choices to protect themselves from COVID-19, the role of public health in providing honest and accurate feedback around the consequences of those choices cannot be overstated. A thrice-yearly booster frequency may have low uptake, but it may provide better protection for those who seek to avoid SARS-CoV-2 infection. For public health authorities to be able to make such recommendations is key—the design of clinical trials to support these types of recommendations is a potentially fruitful avenue for public health researchers to pursue (and one that we are currently pursuing).

As a closely related point, our work suggests that repeat boosting is an important tool for maintaining vaccine efficacy between variant-specific vaccine updates, even though new variants often evade existing vaccines to some extent. An additional dose of a partially matched booster still provides additional protection against new variants, increasing the likelihood of a superior outcome relative to not taking the dose.

Third, our work suggests the importance of continuing to support alternative dose routes, such as intranasal vaccines, which may possess a more favorable efficacy-to-toxicity ratio (“therapeutic index”) to support more frequent dosing. Exploring alternative dose routes with superior therapeutic indices can also allow for a more complete assessment of alternative boosting frequencies. For example, a weak mucosal immune response from one or two doses of an intranasal vaccine may be compensated for by additional doses if the intranasal vaccine has a higher therapeutic index.

Our work also suggests that nAb persistence is worth optimizing. Hypothetical vaccines inducing more durable nAbs provided better protection and were more likely to lead to the elimination of SARS-CoV-2. Future vaccine development work should focus on nAb durability and interindividual variability as a differentiating factor in the target product profile. This objective may be achievable by changing adjuvants, vaccine formulations, dose, and/or schedule, for example. Preclinical-to-clinical projections of antibody persistence can be used at the discovery stage. Moving into the clinic, the same projections may be used in a Bayesian framework to design parsimonious clinical trials focused on clinical pharmacokinetics to quantify nAb durability in patients and how this varies between individuals. Additionally, interindividual variability in nAb persistence may be shored up by identifying and targeting boosting toward individuals with poor protection [54]. This type of targeting would benefit by the existence of standardized and validated ELISA-like clinical lab tests for nAb titer, which is another potential area of focus for public health agencies.

Finally, our work suggests that the suppression of SARS-CoV-2 transmission may remain within the realm of possibility. Contrary to perceptions on the topic, improved vaccine design and use may permit the suppression of SARS-CoV-2, both for individuals seeking protection from infection and for the population as a whole. Repeatedly dosed vaccines with a longer half-life, a better tolerability profile, or both may allow nAb levels to build up to a point where VE_i_ is maintained even in the face of rapid viral immune evasion. Such a scenario would place the suppression of SARS-CoV-2 within reach, particularly if the vaccines are used in conjunction with other measures such as testing, improvements in air quality, and masking.

As we identify a number of open immunological questions, our work suggests that answering these questions should be a focus of research in the near term. In particular, understanding the impact of long-term repeated boosting on nAb production as well as vaccine side-effect profiles is crucial for enabling more effective use of the existing vaccines. In this context, it bears mentioning that, in the United States as of December 2022, four doses have been recommended for adults in 18 months [223]. Millions of adults in the US have taken vaccine doses on this schedule, and it appears well tolerated. This frequency of vaccine dosing (roughly once every 4 months) is close to the frequency suggested by our work (once every 3 months).

Our work suggests that repeated vaccination is a crucial driver of public health outcomes, but repeated boosting with mRNA vaccines may present tremendous logistical hurdles on a global basis. More readily scaled and distributed technologies (such as spike protein-based vaccines or inactivated vaccines) may also provide acceptable outcomes if patients can tolerate a dosing frequency that is sufficient to enable nAb buildup to the protective levels required to restore VE_s_ (and potentially VE_i_). The side-effect profile of targeted dose routes (such as for intranasal vaccines) should also be examined closely in this context. While manufacturing, tolerability, and compliance constraints may make frequent boosting hard to achieve with the current vaccines, next-generation vaccines should be designed with a target product profile of repeated dosing in mind. For example, room-temperature-stable, nasally administered vaccines based on low-cost technologies would make it easier for us to achieve the goal of widespread and repeated vaccine coverage.

## 5. Conclusions

Taken together, our findings suggest that there are ways to improve the utility of the current crop of vaccines in controlling SARS-CoV-2. Our work suggests areas of research focus on open immunological questions that must be resolved in order to establish proof-of-concept for such a strategy. Our work also suggests a few key properties that the next generation of vaccines should take into account: the durability of nAb response, the tolerability of repeat dosing, and manufacturability at scale.

While it is often said that “learning to live with the virus” is inevitable, our work suggests that this is not the case. Improving the performance of existing vaccines and rationally designing future vaccines hold the key to restoring the promise of vaccine control of SARS-CoV-2.

## Figures and Tables

**Table 1:** Parameter values for the fitted pharmacokinetic model describing nAb kinetics. Parameter values for fitted nAb kinetics model with standard error (SE) and relative standard error (RSE), where corr_k,Tin_ is the correlation between k_p_ and T_in_, and a is the coefficient of constant error.

Parameter	Value	Units	Standard Error	Relative Standard Error (%)
*Fixed effects (median)*				
k_p, pop_	38.3	IC50/days	5.31	13.9
k_el, pop_	0.00628	1/days	0.0012	19.1
T_in, pop_	26.9	days	26.9	15.1
*Standard deviation of the random effects*				
ω_k_	0.731	IC50/days	0.156	21.3
ω_kel_	0.346	1/days	0.141	40.8
ω_Tin_	0.708	days	0.181	25.6
*Correlations*				
corr_k,Tin_	−0.977		0.0255	2.61
*Error model parameters*				
a	121		9.11	7.51

**Table 2:** Agent-based model parameters.

Parameter	Value	Source
h (Hill coefficient)	1.018	[32]
EC_50,_*_infection_* (CP titer *)	0.35	
EC_50,_*_death_* (CP titer *)	0.03	[32]
R_0_ (individuals)	8.2	[76,77]
Immune evasion half-life (days)	73	[78]
Infection duration (days)	10	[70]
Increase in titer on reinfection (fold)	14.4	[79]
Increase in titer on revaccination (fold)	10	[71,80,81]
Revaccination interval (days)	60–365	[82]
Fraction boosted (%)	50	[83]
Simulation duration (years)	1 and 10	
Simulated population size	100,000	

* Expressed as a fraction of peak neutralizing antibody titer in COVID-19 convalescents.

**Table 3:** Scenario-specific parameter values for SIRS model.

Parameter	Value	Units
Natural immunity decay rate	0.002 [57]	1/days
Vaccine immunity decay rate	0.002 [57]	1/days
Delta R_0_	6.0 [84]	Individuals
Alpha R_0_	5.3 [85]	Individuals
Vei, delta, recent primary series	70.2 [5]	%
Vei, delta, distant primary series	40.2 [5]	%
Vei, delta, recent first booster	93 [86]	%
Vei, pre-delta, primary series	90 [87]	%
Vei, pre-delta, booster	95	%
Cross-immunity protection	81 [88,89]	%
Percentage of adults in US population	78 [90]	%
US population vaccinated, summer 2021	48 [91]	%
Transmission mitigation, summer 2021	50	%

R_0_: intrinsic reproductive number; Vei: vaccine efficacy against infection; cross-immunity protection: protection against one strain conferred by infection by the other (assumed in this paper to be symmetric).

## Data Availability

The data used to build the models in this paper were taken from the public domain. Code is available upon request. This is a mathematical modeling study, so no physical materials were used. All correspondence is to be directed to A.C.

## Supplementary Materials

The following supporting information can be downloaded at https://www.mdpi.com/article/10.3390/vaccines13040368/s1: Section S1: Lack of evidence for a protective role for T cell immunity for SARS-CoV-2; Table S1: Reported immune escape mutations in the T cell epitopes of the spike protein, with their current frequency in the cov-spectrum database (as of 01/19/23); Figure S1: ROC curve describing spike-specific T cell frequency as a predictor of testing positive for SARS-CoV-2; Figure S2: Goodness-of-fit assessment for nAb kinetics model describing antibody decay post-COVID-19 infection; Figure S3: Mixed-effects model fit to IgG kinetics after SARS-CoV-2 infection; Figure S4: more frequent vaccination schedule using current vaccines may benefit individuals with the shortest vaccine nAb half-lives; Figure S5: A more frequent vaccination schedule using current vaccines may benefit individuals with the shortest vaccine nAb half-lives; Figure S6: Increased durability of vaccine nAb titers improves population-level COVID-19 outcomes; Figure S7: Boosters in the summer of 2021 could have blunted the impact of the delta wave in the US, even if greater adult compliance could not be achieved; Figure S8: Impact of twice-yearly boosters on COVID-19 outcomes; Figure S9: Impact of thrice-yearly boosters on COVID-19 outcomes; Figure S10: Impact of Moderna boosters on individual COVID-19 outcomes in the event of negligible booster uptake.

## References

[1] Baden L.R., El Sahly H.M., Essink B., Kotloff K., Frey S., Novak R., Diemert D., Spector S.A., Rouphael N., Creech C.B. (2021). Efficacy and Safety of the mRNA-1273 SARS-CoV-2 Vaccine. N. Eng. J. Med..

[2] Tenforde M.W., Olson S.M., Self W.H., Talbot H.K., Lindsell C.J., Steingrub J.S., Shapiro N.I., Ginde A.A., Douin D.J., Prekker M.E. (2021). Effectiveness of Pfizer-BioNTech and Moderna Vaccines Against COVID-19 Among Hospitalized Adults Aged ≥65 Years—United States, January–March 2021. MMWR Morb. Mortal. Wkly. Rep..

[3] Pfizer and BioNTech Conclude Phase 3 Study of COVID-19 Vaccine Candidate, Meeting All Primary Efficacy Endpoints. https://www.pfizer.com/news/press-release/press-release-detail/pfizer-and-biontech-conclude-phase-3-study-covid-19-vaccine.

[4] Tseng H.F., Ackerson B.K., Luo Y., Sy L.S., Talarico C.A., Tian Y., Bruxvoort K.J., Tubert J.E., Florea A., Ku J.H. (2022). Effectiveness of mRNA-1273 against SARS-CoV-2 Omicron and Delta Variants. Nat. Med..

[5] Prunas O., Warren J.L., Crawford F.W., Gazit S., Patalon T., Weinberger D.M., Pitzer V.E. (2022). Vaccination with BNT162b2 Reduces Transmission of SARS-CoV-2 to Household Contacts in Israel. Science.

[6] Pegu A., O’Connell S.E., Schmidt S.D., O’Dell S., Talana C.A., Lai L., Albert J., Anderson E., Bennett H., Corbett K.S. (2021). Durability of mRNA-1273 Vaccine-Induced Antibodies against SARS-CoV-2 Variants. Science.

[7] Ibarrondo F.J., Hofmann C., Fulcher J.A., Goodman-Meza D., Mu W., Hausner M.A., Ali A., Balamurugan A., Taus E., Elliott J. (2021). Primary, Recall, and Decay Kinetics of SARS-CoV-2 Vaccine Antibody Responses. ACS Nano.

[8] Levin E.G., Lustig Y., Cohen C., Fluss R., Indenbaum V., Amit S., Doolman R., Asraf K., Mendelson E., Ziv A. (2021). Waning Immune Humoral Response to BNT162b2 Covid-19 Vaccine over 6 Months. N. Engl. J. Med..

[9] Notarte K.I., Guerrero-Arguero I., Velasco J.V., Ver A.T., Santos de Oliveira M.H., Catahay J.A., Khan M.S.R., Pastrana A., Juszczyk G., Torrelles J.B. (2022). Characterization of the Significant Decline in Humoral Immune Response Six Months Post-SARS-CoV-2 mRNA Vaccination: A Systematic Review. J. Med. Virol..

[10] Weisblum Y., Schmidt F., Zhang F., DaSilva J., Poston D., Lorenzi J.C., Muecksch F., Rutkowska M., Hoffmann H.-H., Michailidis E. (2020). Escape from Neutralizing Antibodies by SARS-CoV-2 Spike Protein Variants. eLife.

[11] Li Q., Wu J., Nie J., Zhang L., Hao H., Liu S., Zhao C., Zhang Q., Liu H., Nie L. (2020). The Impact of Mutations in SARS-CoV-2 Spike on Viral Infectivity and Antigenicity. Cell.

[12] Greaney A.J., Loes A.N., Crawford K.H.D., Starr T.N., Malone K.D., Chu H.Y., Bloom J.D. (2021). Comprehensive Mapping of Mutations in the SARS-CoV-2 Receptor-Binding Domain That Affect Recognition by Polyclonal Human Plasma Antibodies. Cell Host Microbe.

[13] Andreano E., Piccini G., Licastro D., Casalino L., Johnson N.V., Paciello I., Dal Monego S., Pantano E., Manganaro N., Manenti A. (2021). SARS-CoV-2 Escape from a Highly Neutralizing COVID-19 Convalescent Plasma. Proc. Natl. Acad. Sci. USA.

[14] Watson O.J., Barnsley G., Toor J., Hogan A.B., Winskill P., Ghani A.C. (2022). Global Impact of the First Year of COVID-19 Vaccination: A Mathematical Modelling Study. Lancet Infect. Dis..

[15] Björk J., Bonander C., Moghaddassi M., Rasmussen M., Malmqvist U., Inghammar M., Kahn F. (2022). COVID-19 Vaccine Effectiveness against Severe Disease from SARS-CoV-2 Omicron BA.1 and BA.2 Subvariants–Surveillance Results from Southern Sweden, December 2021 to March 2022. Eurosurveillance.

[16] COVID-19 Vaccine Surveillance Report: Week 15. https://assets.publishing.service.gov.uk/government/uploads/system/uploads/attachment_data/file/1069256/Vaccine_surveillance_report_-_week_15.pdf.

[17] Suah J.L., Husin M., Tok P.S.K., Tng B.H., Thevananthan T., Low E.V., Appannan M.R., Zin F.M., Zin S.M., Yahaya H. (2022). Waning COVID-19 Vaccine Effectiveness for BNT162b2 and CoronaVac in Malaysia: An Observational Study. Int. J. Infect. Dis..

[18] Wright B.J., Tideman S., Diaz G.A., French T., Parsons G.T., Robicsek A. (2022). Comparative Vaccine Effectiveness against Severe COVID-19 over Time in US Hospital Administrative Data: A Case-Control Study. Lancet Respir. Med..

[19] Immunogenicity of BA.5 Bivalent mRNA Vaccine Boosters|NEJM. https://www.nejm.org/doi/full/10.1056/NEJMc2213948.

[20] Link-Gelles R., Ciesla A.A., Fleming-Dutra K.E., Smith Z.R., Britton A., Wiegand R.E., Miller J.D., Accorsi E.K., Schrag S.J., Verani J.R. (2022). Effectiveness of Bivalent mRNA Vaccines in Preventing Symptomatic SARS-CoV-2 Infection—Increasing Community Access to Testing Program, United States, September-November 2022. MMWR Morb. Mortal. Wkly. Rep..

[21] Cromer D., Steain M., Reynaldi A., Schlub T.E., Wheatley A.K., Juno J.A., Kent S.J., Triccas J.A., Khoury D.S., Davenport M.P. (2022). Neutralising Antibody Titres as Predictors of Protection against SARS-CoV-2 Variants and the Impact of Boosting: A Meta-Analysis. Lancet Microbe.

[22] China Eases ‘Zero Covid’ Restrictions in Victory for Protesters. https://www.nytimes.com/2022/12/07/world/asia/china-zero-covid-protests.html.

[23] CDC Streamlines COVID-19 Guidance to Help the Public Better Protect Themselves and Understand Their Risk. https://archive.cdc.gov/#/details?url=https://www.cdc.gov/media/releases/2022/p0811-covid-guidance.html.

[24] Focosi D. (2022). Molnupiravir: From Hope to Epic Fail?. Viruses.

[25] Pfizer Reports Additional Data on PAXLOVID^TM^ Supporting Upcoming New Drug Application Submission to U.S. FDA. https://www.businesswire.com/news/home/20220613005755/en/Pfizer-Reports-Additional-Data-on-PAXLOVID%E2%84%A2-Supporting-Upcoming-New-Drug-Application-Submission-to-U.S.-FDA.

[26] Coronavirus (COVID-19) Update: FDA Revokes Emergency Use Authorization for Monoclonal Antibody Bamlanivimab. https://www.fda.gov/news-events/press-announcements/coronavirus-covid-19-update-fda-revokes-emergency-use-authorization-monoclonal-antibody-bamlanivimab.

[27] There Are No Useful Monoclonal Antibody Treatments Left Against New COVID Variants. https://www.usnews.com/news/health-news/articles/2022-12-05/there-are-no-useful-monoclonal-antibody-treatments-left-against-new-covid-variants.

[28] Coronavirus (COVID-19) Update: FDA Limits Use of Certain Monoclonal Antibodies to Treat COVID-19 Due to the Omicron Variant. https://www.medpagetoday.com/infectiousdisease/covid19/113392.

[29] Hashem A.M., Algaissi A., Almahboub S.A., Alfaleh M.A., Abujamel T.S., Alamri S.S., Alluhaybi K.A., Hobani H.I., AlHarbi R.H., Alsulaiman R.M. (2020). Early Humoral Response Correlates with Disease Severity and Outcomes in COVID-19 Patients. Viruses.

[30] Maier H.E., Balmaseda A., Ojeda S., Cerpas C., Sanchez N., Plazaola M., van Bakel H., Kubale J., Lopez R., Saborio S. (2021). An Immune Correlate of SARS-CoV-2 Infection and Severity of Reinfections. medRxiv.

[31] Addetia A., Crawford K.H.D., Dingens A., Zhu H., Roychoudhury P., Huang M.-L., Jerome K.R., Bloom J.D., Greninger A.L. (2020). Neutralizing Antibodies Correlate with Protection from SARS-CoV-2 in Humans during a Fishery Vessel Outbreak with a High Attack Rate. J. Clin. Microbiol..

[32] Khoury D.S., Cromer D., Reynaldi A., Schlub T.E., Wheatley A.K., Juno J.A., Subbarao K., Kent S.J., Triccas J.A., Davenport M.P. (2021). Neutralizing Antibody Levels Are Highly Predictive of Immune Protection from Symptomatic SARS-CoV-2 Infection. Nat. Med..

[33] Feng S., Phillips D.J., White T., Sayal H., Aley P.K., Bibi S., Dold C., Fuskova M., Gilbert S.C., Hirsch I. (2021). Correlates of Protection against Symptomatic and Asymptomatic SARS-CoV-2 Infection. Nat. Med..

[34] Gilbert P.B., Montefiori D.C., McDermott A.B., Fong Y., Benkeser D., Deng W., Zhou H., Houchens C.R., Martins K., Jayashankar L. (2022). Immune Correlates Analysis of the mRNA-1273 COVID-19 Vaccine Efficacy Clinical Trial. Science.

[35] Cohen J., Stuart R., Rosenfeld K., Lyons H., White M., Kerr C., Klein D., Famulare M. (2021). Quantifying the Role of Naturally- and Vaccine-Derived Neutralizing Antibodies as a Correlate of Protection against COVID-19 Variant. medRxiv.

[36] Cele S., Jackson L., Khoury D.S., Khan K., Moyo-Gwete T., Tegally H., San J.E., Cromer D., Scheepers C., Amoako D.G. (2022). Omicron Extensively but Incompletely Escapes Pfizer BNT162b2 Neutralization. Nature.

[37] Cromer D., Reynaldi A., Steain M., Triccas J.A., Davenport M.P., Khoury D.S. (2022). Relating In Vitro Neutralization Level and Protection in the CVnCoV (CUREVAC) Trial. Clin. Infect. Dis..

[38] Koutsakos M., Lee W.S., Reynaldi A., Tan H.-X., Gare G., Kinsella P., Liew K.C., Taiaroa G., Williamson D.A., Kent H.E. (2022). The Magnitude and Timing of Recalled Immunity after Breakthrough Infection Is Shaped by SARS-CoV-2 Variants. Immunity.

[39] The T-Cell Covid Cavalry. https://www.wsj.com/articles/the-t-cell-covid-cavalry-antibodies-vaccines-omicron-11640906490.

[40] Leslie M. (2020). T Cells Found in Coronavirus Patients “bode Well” for Long-Term Immunity. Science.

[41] T Cells Protect against COVID-19 in Absence of Antibody Response. https://www.nih.gov/news-events/nih-research-matters/t-cells-protect-against-covid-19-absence-antibody-response.

[42] Tarke A., Coelho C.H., Zhang Z., Dan J.M., Yu E.D., Methot N., Bloom N.I., Goodwin B., Phillips E., Mallal S. (2022). SARS-CoV-2 Vaccination Induces Immunological T Cell Memory Able to Cross-Recognize Variants from Alpha to Omicron. Cell.

[43] Dan J.M., Mateus J., Kato Y., Hastie K.M., Yu E.D., Faliti C.E., Grifoni A., Ramirez S.I., Haupt S., Frazier A. (2021). Immunological Memory to SARS-CoV-2 Assessed for up to 8 Months after Infection. Science.

[44] Guo L., Wang G., Wang Y., Zhang Q., Ren L., Gu X., Huang T., Zhong J., Wang Y., Wang X. (2022). SARS-CoV-2-Specific Antibody and T-Cell Responses 1 Year after Infection in People Recovered from COVID-19: A Longitudinal Cohort Study. Lancet Microbe.

[45] Tenforde M.W. (2022). Early Estimates of Bivalent mRNA Vaccine Effectiveness in Preventing COVID-19–Associated Emergency Department or Urgent Care Encounters and Hospitalizations Among Immunocompetent Adults—VISION Network, Nine States, September–November 2022. MMWR Morb. Mortal. Wkly. Rep..

[46] Polack F.P., Thomas S.J., Kitchin N., Absalon J., Gurtman A., Lockhart S., Perez J.L., Pérez Marc G., Moreira E.D., Zerbini C. (2020). Safety and Efficacy of the BNT162b2 mRNA Covid-19 Vaccine. N. Engl. J. Med..

[47] COVID-19 SeroHub. https://covid19serohub.nih.gov/.

[48] EXCLUSIVE WHO Estimates COVID-19 Boosters Needed Yearly for Most Vulnerable. https://www.reuters.com/business/healthcare-pharmaceuticals/exclusive-who-estimates-covid-19-boosters-needed-yearly-most-vulnerable-2021-06-24/.

[49] Oliver S. Evidence to Recommendation Framework; COVID-19 Vaccination Guidance. Proceedings of the ACIP Meeting.

[50] Weekly COVID-19 Vaccination Dashboard|COVIDVaxView|CDC. https://www.cdc.gov/covidvaxview/weekly-dashboard/.

[51] Acko T. COVID-19 Booster Dose in India: Eligibility & Registration. https://www.acko.com/health-insurance/covid-19-booster-dose-in-india/.

[52] Current Rules and Recommendations. https://www.krisinformation.se/en/hazards-and-risks/disasters-and-incidents/2020/official-information-on-the-new-coronavirus/current-rules-and-recommendations.

[53] Who’s Eligible for the 2024 COVID-19 Vaccine, or ‘Autumn Booster’?—UK Health Security Agency. https://ukhsa.blog.gov.uk/2024/08/02/whos-eligible-for-the-2024-covid-19-vaccine-or-autumn-booster/.

[54] Stoddard M., Yuan L., Sarkar S., Mangalaganesh S., Nolan R.P., Bottino D., Hather G., Hochberg N.S., White L.F., Chakravarty A. (2023). Heterogeneity in Vaccinal Immunity to SARS-CoV-2 Can Be Addressed by a Personalized Booster Strategy. Vaccines.

[55] Mantovani A., Morrone M.C., Patrono C., Santoro M.G., Schiaffino S., Remuzzi G., Bussolati G. (2022). COVID-19 Commission of the Accademia Nazionale dei Lincei Long Covid: Where We Stand and Challenges Ahead. Cell Death Differ..

[56] Al-Aly Z., Bowe B., Xie Y. (2022). Long COVID after Breakthrough SARS-CoV-2 Infection. Nat. Med..

[57] Stoddard M., Yuan L., Sarkar S., Mazewski M., van Egeren D., Mangalaganesh S., Nolan R.P., Rogers M.S., Hather G., White L.F. (2023). Shielding under Endemic SARS-CoV-2 Conditions Is Easier Said than Done: A Model-Based Analysis. medRxiv.

[58] New Data Shows Long Covid Is Keeping as Many as 4 Million People out of Work. https://www.brookings.edu/articles/new-data-shows-long-covid-is-keeping-as-many-as-4-million-people-out-of-work/.

[59] Long-Haulers and Labor Market Outcomes. https://www.minneapolisfed.org/research/institute-working-papers/long-haulers-and-labor-market-outcomes.

[60] Notarte K.I., Catahay J.A., Velasco J.V., Pastrana A., Ver A.T., Pangilinan F.C., Peligro P.J., Casimiro M., Guerrero J.J., Gellaco M.M.L. (2022). Impact of COVID-19 Vaccination on the Risk of Developing Long-COVID and on Existing Long-COVID Symptoms: A Systematic Review. EClinicalMedicine.

[61] Azzolini E., Levi R., Sarti R., Pozzi C., Mollura M., Mantovani A., Rescigno M. (2022). Association Between BNT162b2 Vaccination and Long COVID After Infections Not Requiring Hospitalization in Health Care Workers. JAMA.

[62] Gao P., Liu J., Liu M. (2022). Effect of COVID-19 Vaccines on Reducing the Risk of Long COVID in the Real World: A Systematic Review and Meta-Analysis. Int. J. Environ. Res. Public Health.

[63] Watanabe A., Iwagami M., Yasuhara J., Takagi H., Kuno T. (2023). Protective Effect of COVID-19 Vaccination against Long COVID Syndrome: A Systematic Review and Meta-Analysis. Vaccine.

[64] Man M.A., Rosca D., Bratosin F., Fira-Mladinescu O., Ilie A.C., Burtic S.-R., Fildan A.P., Fizedean C.M., Jianu A.M., Negrean R.A. (2024). Impact of Pre-Infection COVID-19 Vaccination on the Incidence and Severity of Post-COVID Syndrome: A Systematic Review and Meta-Analysis. Vaccines.

[65] Kim S., Kang H. (2022). Is the Vaccine for COVID-19 Effective in Preventing and Treating Long COVID?. J. Stud. Res..

[66] Wang K., Long Q.-X., Deng H.-J., Hu J., Gao Q.-Z., Zhang G.-J., He C.-L., Huang L.-Y., Hu J.-L., Chen J. (2021). Longitudinal Dynamics of the Neutralizing Antibody Response to Severe Acute Respiratory Syndrome Coronavirus 2 (SARS-CoV-2) Infection. Clin. Infect. Dis..

[67] Leung K., Jit M., Lau E.H.Y., Wu J.T. (2017). Social Contact Patterns Relevant to the Spread of Respiratory Infectious Diseases in Hong Kong. Sci. Rep..

[68] United States Demographic Statistics. https://www.infoplease.com/us/census/demographic-statistics.

[69] SARS-CoV-2 Infection Rates of Antibody-Positive Compared with Antibody-Negative Health-Care Workers in England: A Large, Multicentre, Prospective Cohort Study (SIREN)—The Lancet. https://www.thelancet.com/article/S0140-6736(21)00675-9/fulltext.

[70] Rocklöv J., Sjödin H., Wilder-Smith A. (2020). COVID-19 Outbreak on the Diamond Princess Cruise Ship: Estimating the Epidemic Potential and Effectiveness of Public Health Countermeasures. J. Travel Med..

[71] Regev-Yochay G., Gonen T., Gilboa M., Mandelboim M., Indenbaum V., Amit S., Meltzer L., Asraf K., Cohen C., Fluss R. (2022). Efficacy of a Fourth Dose of Covid-19 mRNA Vaccine against Omicron. N. Engl. J. Med..

[72] Khan K., Karim F., Cele S., Reedoy K., San J.E., Lustig G., Tegally H., Rosenberg Y., Bernstein M., Jule Z. (2022). Omicron Infection Enhances Delta Antibody Immunity in Vaccinated Persons. Nature.

[73] Levin A.T., Hanage W.P., Owusu-Boaitey N., Cochran K.B., Walsh S.P., Meyerowitz-Katz G. (2020). Assessing the Age Specificity of Infection Fatality Rates for COVID-19: Systematic Review, Meta-Analysis, and Public Policy Implications. Eur. J. Epidemiol..

[74] Liu Y., Yu Y., Zhao Y., He D. (2022). Reduction in the Infection Fatality Rate of Omicron Variant Compared with Previous Variants in South Africa. Int. J. Infect. Dis..

[75] Van Egeren D., Stoddard M., Novokhodko A., Rogers M.S., Joseph-McCarthy D., Zetter B., Chakravarty A. (2021). Rapid Relaxation of Pandemic Restrictions after Vaccine Rollout Favors Growth of SARS-CoV-2 Variants: A Model-Based Analysis. PLoS ONE.

[76] Liu Y., Rocklöv J. (2022). The Effective Reproductive Number of the Omicron Variant of SARS-CoV-2 Is Several Times Relative to Delta. J. Travel Med..

[77] Du Z., Hong H., Wang S., Ma L., Liu C., Bai Y., Adam D.C., Tian L., Wang L., Lau E.H.Y. (2022). Reproduction Number of the Omicron Variant Triples That of the Delta Variant. Viruses.

[78] Cao Y., Jian F., Wang J., Yu Y., Song W., Yisimayi A., Wang J., An R., Chen X., Zhang N. (2022). Imprinted SARS-CoV-2 Humoral Immunity Induces Convergent Omicron RBD Evolution. Nature.

[79] Khan K., Karim F., Cele S., San J.E., Lustig G., Bernstein M., Ganga Y., Jule Z., Reedoy K., Ngcobo N. (2022). 2 Omicron Infection Enhances Neutralizing Immunity against the Delta Variant. medRxiv.

[80] Hein S., Mhedhbi I., Zahn T., Sabino C., Benz N.I., Husria Y., Renelt P.M., Braun F., Oberle D., Maier T.J. (2022). Quantitative and Qualitative Difference in Antibody Response against Omicron and Ancestral SARS-CoV-2 after Third and Fourth Vaccination. Vaccines.

[81] Chu L., Vrbicky K., Montefiori D., Huang W., Nestorova B., Chang Y., Carfi A., Edwards D.K., Oestreicher J., Legault H. (2022). Immune Response to SARS-CoV-2 after a Booster of mRNA-1273: An Open-Label Phase 2 Trial. Nat. Med..

[82] Joseph A. White House Signals Most People Will Only Need Annual Covid Booster. STAT.

[83] CDC COVID Data Tracker Weekly Review. https://archive.cdc.gov/#/details?url=https://www.cdc.gov/coronavirus/2019-ncov/covid-data/covidview/past-reports/index.html.

[84] Fisman D.N., Tuite A.R. (2021). Evaluation of the Relative Virulence of Novel SARS-CoV-2 Variants: A Retrospective Cohort Study in Ontario, Canada. CMAJ.

[85] Davies N.G., Abbott S., Barnard R.C., Jarvis C.I., Kucharski A.J., Munday J.D., Pearson C.A.B., Russell T.W., Tully D.C., Washburne A.D. (2021). Estimated Transmissibility and Impact of SARS-CoV-2 Lineage B.1.1.7 in England. Science.

[86] Barda N., Dagan N., Cohen C., Hernán M.A., Lipsitch M., Kohane I.S., Reis B.Y., Balicer R.D. (2021). Effectiveness of a Third Dose of the BNT162b2 mRNA COVID-19 Vaccine for Preventing Severe Outcomes in Israel: An Observational Study. Lancet.

[87] Feikin D.R., Higdon M.M., Abu-Raddad L.J., Andrews N., Araos R., Goldberg Y., Groome M.J., Huppert A., O’Brien K.L., Smith P.G. (2022). Duration of Effectiveness of Vaccines against SARS-CoV-2 Infection and COVID-19 Disease: Results of a Systematic Review and Meta-Regression. Lancet.

[88] Helfand M., Fiordalisi C., Wiedrick J., Ramsey K.L., Armstrong C., Gean E., Winchell K., Arkhipova-Jenkins I. (2022). Risk for Reinfection After SARS-CoV-2: A Living, Rapid Review for American College of Physicians Practice Points on the Role of the Antibody Response in Conferring Immunity Following SARS-CoV-2 Infection. Ann. Intern. Med..

[89] SARS-CoV-2 Variants of Concern and Variants under Investigation in England—Technical Briefing 28. https://assets.publishing.service.gov.uk/government/uploads/system/uploads/attachment_data/file/1033101/Technical_Briefing_28_12_Nov_2021.pdf.

[90] Ogunwole S.U., Rabe M.A., Roberts A.W., Caplan Z. Adult Population Grew Faster Than Total Population From 2010 to 2020. https://www.census.gov/library/stories/2021/08/united-states-adult-population-grew-faster-than-nations-total-population-from-2010-to-2020.html.

[91] Mathieu E., Ritchie H., Rodés-Guirao L., Appel C., Giattino C., Hasell J., Macdonald B., Dattani S., Beltekian D., Ortiz-Ospina E. (2020). Coronavirus Pandemic (COVID-19). Our World in Data. https://ourworldindata.org/coronavirus.

[92] Stoddard M., Novokhodko A., Sarkar S., Van Egeren D., White L.F., Hochberg N.S., Rogers M.S., Zetter B., Joseph-McCarthy D., Chakravarty A. (2022). Endemicity Is Not a Victory: The Unmitigated Downside Risks of Widespread SARS-CoV-2 Transmission. COVID.

[93] Bottino D., Hather G., Yuan L., Stoddard M., White L., Chakravarty A. (2021). Using Mixed-Effects Modeling to Estimate Decay Kinetics of Response to SARS-CoV-2 Infection. Antib. Ther..

[94] Delta Variant Already Dominant in U.S. CDC Estimates Show. https://www.reuters.com/world/us/delta-variant-already-dominant-us-cdc-estimates-show-2021-07-07/.

[95] COVID Data Tracker. https://covid.cdc.gov/covid-data-tracker.

[96] United States COVID—Coronavirus Statistics. https://www.worldometers.info/coronavirus/country/us/.

[97] Cases, Data, and Surveillance. https://archive.cdc.gov/#/details?url=https://www.cdc.gov/coronavirus/2019-ncov/covid-data/covidview/past-reports/03182022.html.

[98] Government of Canada S.C. Experiences of Canadians with Long-Term Symptoms Following COVID-19. https://www150.statcan.gc.ca/n1/pub/75-006-x/2023001/article/00015-eng.htm.

[99] Van Egeren D., Novokhodko A., Stoddard M., Tran U., Zetter B., Rogers M., Pentelute B.L., Carlson J.M., Hixon M., Joseph-McCarthy D. (2021). Risk of Rapid Evolutionary Escape from Biomedical Interventions Targeting SARS-CoV-2 Spike Protein. PLoS ONE.

[100] Stoddard M., Sarkar S., Yuan L., Nolan R.P., White D.E., White L.F., Hochberg N.S., Chakravarty A. (2021). Beyond the New Normal: Assessing the Feasibility of Vaccine-Based Suppression of SARS-CoV-2. PLoS ONE.

[101] Egeren D.V., Novokhodko A., Stoddard M., Tran U., Zetter B., Rogers M., Pentelute B.L., Carlson J.M., Hixon M., Joseph-McCarthy D. (2020). Risk of Evolutionary Escape from Neutralizing Antibodies Targeting SARS-CoV-2 Spike Protein. medRxiv.

[102] Stoddard M., Van Egeren D., Johnson K.E., Rao S., Furgeson J., White D.E., Nolan R.P., Hochberg N., Chakravarty A. (2021). Individually Optimal Choices Can Be Collectively Disastrous in COVID-19 Disease Control. BMC Public Health.

[103] Alejo J.L., Mitchell J., Chiang T.P.-Y., Abedon A.T., Boyarsky B.J., Avery R.K., Tobian A.A.R., Levan M.L., Massie A.B., Garonzik-Wang J.M. (2021). Antibody Response to a Fourth Dose of a SARS-CoV-2 Vaccine in Solid Organ Transplant Recipients: A Case Series. Transplantation.

[104] Caillard S., Thaunat O., Benotmane I., Masset C., Blancho G. (2022). Antibody Response to a Fourth Messenger RNA COVID-19 Vaccine Dose in Kidney Transplant Recipients: A Case Series. Ann. Intern. Med..

[105] Abedon A.T., Teles M.S., Alejo J.L., Kim J.D., Mitchell J., Chiang T.P.Y., Avery R.K., Tobian A.A.R., Levan M.L., Warren D.S. (2022). Improved Antibody Response After a Fifth Dose of a SARS-CoV-2 Vaccine in Solid Organ Transplant Recipients: A Case Series. Transplantation.

[106] Parisi S.G., Mengoli C., Basso M., Vicenti I., Gatti F., Scaggiante R., Fiaschi L., Giammarino F., Iannetta M., Malagnino V. (2022). Long-Term Longitudinal Analysis of Neutralizing Antibody Response to Three Vaccine Doses in a Real-Life Setting of Previously SARS-CoV-2 Infected Healthcare Workers: A Model for Predicting Response to Further Vaccine Doses. Vaccines.

[107] Teles M., Connolly C.M., Frey S., Chiang T.P.-Y., Alejo J.J., Boyarsky B.J., Shah A.A., Albayda J., Christopher-Stine L., Werbel W.A. (2022). Attenuated Response to Fourth Dose SARS-CoV-2 Vaccination in Patients with Autoimmune Disease: A Case Series. Ann. Rheum. Dis..

[108] Gao F.-X., Wu R.-X., Shen M.-Y., Huang J.-J., Li T.-T., Hu C., Luo F.-Y., Song S.-Y., Mu S., Hao Y.-N. (2022). Extended SARS-CoV-2 RBD Booster Vaccination Induces Humoral and Cellular Immune Tolerance in Mice. IScience.

[109] Sellers R.S. (2017). Translating Mouse Models: Immune Variation and Efficacy Testing. Toxicol. Pathol..

[110] Chemaitelly H., Ayoub H.H., Tang P., Hasan M.R., Coyle P., Yassine H.M., Al-Khatib H.A., Smatti M.K., Al-Kanaani Z., Al-Kuwari E. (2022). Immune Imprinting and Protection against Repeat Reinfection with SARS-CoV-2. N. Engl. J. Med..

[111] Collier A.Y., Miller J., Hachmann N.P., McMahan K., Liu J., Bondzie E.A., Gallup L., Rowe M., Schonberg E., Thai S. (2023). Immunogenicity of BA.5 Bivalent mRNA Vaccine Boosters. N. Engl. J. Med..

[112] Qu P., Faraone J.N., Evans J.P., Zheng Y.-M., Carlin C., Anghelina M., Stevens P., Fernandez S., Jones D., Panchal A. (2023). Extraordinary Evasion of Neutralizing Antibody Response by Omicron XBB.1.5, CH.1.1 and CA.3.1 Variants. bioRxiv.

[113] Gilboa M., Regev-Yochay G., Mandelboim M., Indenbaum V., Asraf K., Fluss R., Amit S., Mendelson E., Doolman R., Afek A. (2022). Durability of Immune Response After COVID-19 Booster Vaccination and Association With COVID-19 Omicron Infection. JAMA Netw. Open.

[114] Bellusci L., Grubbs G., Zahra F.T., Forgacs D., Golding H., Ross T.M., Khurana S. (2022). Antibody Affinity and Cross-Variant Neutralization of SARS-CoV-2 Omicron BA.1, BA.2 and BA.3 Following Third mRNA Vaccination. Nat. Commun..

[115] Lau C.S., Oh M.L.H., Phua S.K., Liang Y.-L., Aw T.C. (2022). 210-Day Kinetics of Total, IgG, and Neutralizing Spike Antibodies across a Course of 3 Doses of BNT162b2 mRNA Vaccine. Vaccines.

[116] Liang X.-M., Xu Q.-Y., Jia Z.-J., Wu M.-J., Liu Y.-Y., Lin L.-R., Liu L.-L., Yang T.-C. (2022). A Third Dose of an Inactivated Vaccine Dramatically Increased the Levels and Decay Times of Anti-SARS-CoV-2 Antibodies, but Disappointingly Declined Again: A Prospective, Longitudinal, Cohort Study at 18 Serial Time Points Over 368 Days. Front. Immunol..

[117] Dapporto F., Marchi S., Leonardi M., Piu P., Lovreglio P., Decaro N., Buonvino N., Stufano A., Lorusso E., Bombardieri E. (2022). Antibody Avidity and Neutralizing Response against SARS-CoV-2 Omicron Variant after Infection or Vaccination. J. Immunol. Res..

[118] Plotkin S.A. (2010). Correlates of Protection Induced by Vaccination. Clin. Vaccine Immunol..

[119] Bruel T., Pinaud L., Tondeur L., Planas D., Staropoli I., Porrot F., Guivel-Benhassine F., Attia M., Pelleau S., Woudenberg T. (2022). Neutralising Antibody Responses to SARS-CoV-2 Omicron among Elderly Nursing Home Residents Following a Booster Dose of BNT162b2 Vaccine: A Community-Based, Prospective, Longitudinal Cohort Study. EClinicalMedicine.

[120] Sparks J.A., Wallace Z.S., Seet A.M., Gianfrancesco M.A., Izadi Z., Hyrich K.L., Strangfeld A., Gossec L., Carmona L., Mateus E.F. (2021). Associations of Baseline Use of Biologic or Targeted Synthetic DMARDs with COVID-19 Severity in Rheumatoid Arthritis: Results from the COVID-19 Global Rheumatology Alliance Physician Registry. Ann. Rheum. Dis..

[121] Bitoun S., Henry J., Desjardins D., Vauloup-Fellous C., Dib N., Belkhir R., Mouna L., Joly C., Bitu M., Ly B. (2022). Rituximab Impairs B Cell Response But Not T Cell Response to COVID-19 Vaccine in Autoimmune Diseases. Arthritis Rheumatol..

[122] Andersen K.M., Bates B.A., Rashidi E.S., Olex A.L., Mannon R.B., Patel R.C., Singh J., Sun J., Auwaerter P.G., Ng D.K. (2022). Long-Term Use of Immunosuppressive Medicines and in-Hospital COVID-19 Outcomes: A Retrospective Cohort Study Using Data from the National COVID Cohort Collaborative. Lancet Rheumatol..

[123] Ekin A., Coskun B.N., Dalkilic E., Pehlivan Y. (2023). The Effects of COVID-19 Infection on the Mortality of Patients Receiving Rituximab Therapy. Ir. J. Med. Sci..

[124] Strangfeld A., Schäfer M., Gianfrancesco M.A., Lawson-Tovey S., Liew J.W., Ljung L., Mateus E.F., Richez C., Santos M.J., Schmajuk G. (2021). Factors Associated with COVID-19-Related Death in People with Rheumatic Diseases: Results from the COVID-19 Global Rheumatology Alliance Physician-Reported Registry. Ann. Rheum. Dis..

[125] Avouac J., Drumez E., Hachulla E., Seror R., Georgin-Lavialle S., El Mahou S., Pertuiset E., Pham T., Marotte H., Servettaz A. (2021). COVID-19 Outcomes in Patients with Inflammatory Rheumatic and Musculoskeletal Diseases Treated with Rituximab: A Cohort Study. Lancet Rheumatol..

[126] Sormani M.P., De Rossi N., Schiavetti I., Carmisciano L., Cordioli C., Moiola L., Radaelli M., Immovilli P., Capobianco M., Trojano M. (2021). Disease-Modifying Therapies and Coronavirus Disease 2019 Severity in Multiple Sclerosis. Ann. Neurol..

[127] Bsteh G., Dürauer S., Assar H., Hegen H., Heschl B., Leutmezer F., Pauli F.D., Gradl C., Traxler G., Zulehner G. (2021). Humoral Immune Response after COVID-19 in Multiple Sclerosis: A Nation-Wide Austrian Study. Mult. Scler. J..

[128] Gadani S.P., Reyes-Mantilla M., Jank L., Harris S., Douglas M., Smith M.D., Calabresi P.A., Mowry E.M., Fitzgerald K.C., Bhargava P. (2021). Discordant Humoral and T Cell Immune Responses to SARS-CoV-2 Vaccination in People with Multiple Sclerosis on Anti-CD20 Therapy. medRxiv.

[129] Hada M., Mosholder A.D., Leishear K., Perez-Vilar S. (2022). Systematic Review of Risk of SARS-CoV-2 Infection and Severity of COVID-19 with Therapies Approved to Treat Multiple Sclerosis. Neurol. Sci..

[130] Mohanraj D., Baldwin S., Singh S., Gordon A., Whitelegg A. (2022). Cellular and Humoral Responses to SARS-CoV-2 Vaccination in Immunosuppressed Patients. Cell Immunol..

[131] Calabrese C.M., Kirchner E., Husni E.M., Moss B.P., Fernandez A.P., Jin Y., Calabrese L.H. (2022). Breakthrough SARS-CoV-2 Infections in Patients With Immune-Mediated Disease Undergoing B Cell-Depleting Therapy: A Retrospective Cohort Analysis. Arthritis Rheumatol..

[132] Di Fusco M., Lin J., Vaghela S., Lingohr-Smith M., Nguyen J.L., Scassellati Sforzolini T., Judy J., Cane A., Moran M.M. (2022). COVID-19 Vaccine Effectiveness among Immunocompromised Populations: A Targeted Literature Review of Real-World Studies. Expert Rev. Vaccines.

[133] Mues K.E., Kirk B., Patel D.A., Gelman A., Chavers L.S., Talarico C.A., Esposito D.B., Martin D., Mansi J., Chen X. (2022). Real-World Comparative Effectiveness of mRNA-1273 and BNT162b2 Vaccines among Immunocompromised Adults Identified in Administrative Claims Data in the United States. Vaccine.

[134] Hoff L.S., Ravichandran N., Shinjo S.K., Day J., Sen P., Junior J.G., Lilleker J.B., Joshi M., Agarwal V., Kardes S. (2023). COVID-19 Severity and Vaccine Breakthrough Infections in Idiopathic Inflammatory Myopathies, Other Systemic Autoimmune and Inflammatory Diseases, and Healthy Controls: A Multicenter Cross-Sectional Study from the COVID-19 Vaccination in Autoimmune Diseases (COVAD) Survey. Rheumatol. Int..

[135] Galmiche S., Luong Nguyen L.B., Tartour E., de Lamballerie X., Wittkop L., Loubet P., Launay O. (2022). Immunological and Clinical Efficacy of COVID-19 Vaccines in Immunocompromised Populations: A Systematic Review. Clin. Microbiol. Infect..

[136] Oberhardt V., Luxenburger H., Kemming J., Schulien I., Ciminski K., Giese S., Csernalabics B., Lang-Meli J., Janowska I., Staniek J. (2021). Rapid and Stable Mobilization of CD8+ T Cells by SARS-CoV-2 mRNA Vaccine. Nature.

[137] Jing L., Wu X., Krist M.P., Hsiang T.-Y., Campbell V.L., McClurkan C.L., Favors S.M., Hemingway L.A., Godornes C., Tong D.Q. (2022). T Cell Response to Intact SARS-CoV-2 Includes Coronavirus Cross-Reactive and Variant-Specific Components. JCI Insight.

[138] Moss P. (2022). The T Cell Immune Response against SARS-CoV-2. Nat. Immunol..

[139] Goel R.R., Painter M.M., Apostolidis S.A., Mathew D., Meng W., Rosenfeld A.M., Lundgreen K.A., Reynaldi A., Khoury D.S., Pattekar A. (2021). mRNA Vaccines Induce Durable Immune Memory to SARS-CoV-2 and Variants of Concern. Science.

[140] Mateus J., Dan J.M., Zhang Z., Rydyznski Moderbacher C., Lammers M., Goodwin B., Sette A., Crotty S., Weiskopf D. (2021). Low-Dose mRNA-1273 COVID-19 Vaccine Generates Durable Memory Enhanced by Cross-Reactive T Cells. Science.

[141] Riou C., Keeton R., Moyo-Gwete T., Hermanus T., Kgagudi P., Baguma R., Valley-Omar Z., Smith M., Tegally H., Doolabh D. (2022). Escape from Recognition of SARS-CoV-2 Variant Spike Epitopes but Overall Preservation of T Cell Immunity. Sci. Transl. Med..

[142] Grifoni A., Sidney J., Vita R., Peters B., Crotty S., Weiskopf D., Sette A. (2021). SARS-CoV-2 Human T Cell Epitopes: Adaptive Immune Response against COVID-19. Cell Host Microbe.

[143] Tarke A., Sidney J., Methot N., Yu E.D., Zhang Y., Dan J.M., Goodwin B., Rubiro P., Sutherland A., Wang E. (2021). Impact of SARS-CoV-2 Variants on the Total CD4+ and CD8+ T Cell Reactivity in Infected or Vaccinated Individuals. Cell Rep. Med..

[144] Call in the T-Cell Cavalry to Fight COVID in the Immunocompromised. https://www.medpagetoday.com/opinion/second-opinions/93805.

[145] The Mystery That Could Explain Why COVID Vaccines Work so Well. https://www.smh.com.au/national/the-mystery-that-could-explain-why-covid-vaccines-work-so-well-20210427-p57mq0.html.

[146] Zhang H., Deng S., Ren L., Zheng P., Hu X., Jin T., Tan X. (2021). Profiling CD8+ T Cell Epitopes of COVID-19 Convalescents Reveals Reduced Cellular Immune Responses to SARS-CoV-2 Variants. Cell Rep..

[147] Agerer B., Koblischke M., Gudipati V., Montaño-Gutierrez L.F., Smyth M., Popa A., Genger J.-W., Endler L., Florian D.M., Mühlgrabner V. (2021). SARS-CoV-2 Mutations in MHC-I-Restricted Epitopes Evade CD8+ T Cell Responses. Sci. Immunol..

[148] de Silva T.I., Liu G., Lindsey B.B., Dong D., Moore S.C., Hsu N.S., Shah D., Wellington D., Mentzer A.J., Angyal A. (2021). The Impact of Viral Mutations on Recognition by SARS-CoV-2 Specific T Cells. IScience.

[149] Pretti M.A.M., Galvani R.G., Scherer N.M., Farias A.S., Boroni M. (2022). In Silico Analysis of Mutant Epitopes in New SARS-CoV-2 Lineages Suggest Global Enhanced CD8+ T Cell Reactivity and Also Signs of Immune Response Escape. Infect. Genet. Evol..

[150] Tye E.X.C., Jinks E., Haigh T.A., Kaul B., Patel P., Parry H.M., Newby M.L., Crispin M., Kaur N., Moss P. (2022). Mutations in SARS-CoV-2 Spike Protein Impair Epitope-Specific CD4+ T Cell Recognition. Nat. Immunol..

[151] Dolton G., Rius C., Hasan M.S., Wall A., Szomolay B., Behiry E., Whalley T., Southgate J., Fuller A., COVID-19 Genomics UK (COG-UK) consortium (2022). Emergence of Immune Escape at Dominant SARS-CoV-2 Killer T Cell Epitope. Cell.

[152] Pastorio C., Zech F., Noettger S., Jung C., Jacob T., Sanderson T., Sparrer K.M.J., Kirchhoff F. (2022). Determinants of Spike Infectivity, Processing, and Neutralization in SARS-CoV-2 Omicron Subvariants BA.1 and BA.2. Cell Host Microbe.

[153] Kombe Kombe A.J., Biteghe F.A.N., Ndoutoume Z.N., Jin T. (2022). CD8+ T-Cell Immune Escape by SARS-CoV-2 Variants of Concern. Front. Immunol..

[154] Cohen K.W., Linderman S.L., Moodie Z., Czartoski J., Lai L., Mantus G., Norwood C., Nyhoff L.E., Edara V.V., Floyd K. (2021). Longitudinal Analysis Shows Durable and Broad Immune Memory after SARS-CoV-2 Infection with Persisting Antibody Responses and Memory B and T Cells. medRxiv.

[155] May D.H., Rubin B.E.R., Dalai S.C., Patel K., Shafiani S., Elyanow R., Noakes M.T., Snyder T.M., Robins H.S. (2021). Immunosequencing and Epitope Mapping Reveal Substantial Preservation of the T Cell Immune Response to Omicron Generated by SARS-CoV-2 Vaccines. medRxiv.

[156] Detect and Analyze Variants of SARS-CoV-2. https://cov-spectrum.org.

[157] Ahmed S.F., Quadeer A.A., McKay M.R. (2022). SARS-CoV-2 T Cell Responses Elicited by COVID-19 Vaccines or Infection Are Expected to Remain Robust against Omicron. Viruses.

[158] De Marco L., D’Orso S., Pirronello M., Verdiani A., Termine A., Fabrizio C., Capone A., Sabatini A., Guerrera G., Placido R. (2022). Assessment of T-Cell Reactivity to the SARS-CoV-2 Omicron Variant by Immunized Individuals. JAMA Netw. Open.

[159] Patalon T., Saciuk Y., Peretz A., Perez G., Lurie Y., Maor Y., Gazit S. (2022). Waning Effectiveness of the Third Dose of the BNT162b2 mRNA COVID-19 Vaccine. Nat. Commun..

[160] Levine-Tiefenbrun M., Yelin I., Alapi H., Katz R., Herzel E., Kuint J., Chodick G., Gazit S., Patalon T., Kishony R. (2021). Viral Loads of Delta-Variant SARS-CoV-2 Breakthrough Infections after Vaccination and Booster with BNT162b2. Nat. Med..

[161] Mizrahi B., Lotan R., Kalkstein N., Peretz A., Perez G., Ben-Tov A., Chodick G., Gazit S., Patalon T. (2021). Correlation of SARS-CoV-2-Breakthrough Infections to Time-from-Vaccine. Nat. Commun..

[162] Chemaitelly H., Tang P., Hasan M.R., AlMukdad S., Yassine H.M., Benslimane F.M., Al Khatib H.A., Coyle P., Ayoub H.H., Al Kanaani Z. (2021). Waning of BNT162b2 Vaccine Protection against SARS-CoV-2 Infection in Qatar. N. Engl. J. Med..

[163] Bar-On Y.M., Goldberg Y., Mandel M., Bodenheimer O., Freedman L., Kalkstein N., Mizrahi B., Alroy-Preis S., Ash N., Milo R. (2021). Protection of BNT162b2 Vaccine Booster against Covid-19 in Israel. N. Engl. J. Med..

[164] Lauring A.S., Tenforde M.W., Chappell J.D., Gaglani M., Ginde A.A., McNeal T., Ghamande S., Douin D.J., Talbot H.K., Casey J.D. (2022). Clinical Severity of, and Effectiveness of mRNA Vaccines against, Covid-19 from Omicron, Delta, and Alpha SARS-CoV-2 Variants in the United States: Prospective Observational Study. BMJ.

[165] Dowell A.C., Ireland G., Zuo J., Moss P., Ladhani S., sKIDs Investigation Team (2023). Association of Spike-Specific T Cells With Relative Protection From Subsequent SARS-CoV-2 Omicron Infection in Young Children. JAMA Pediatr..

[166] Hajian-Tilaki K. (2013). Receiver Operating Characteristic (ROC) Curve Analysis for Medical Diagnostic Test Evaluation. Caspian J. Intern. Med..

[167] Pardieck I.N., van der Sluis T.C., van der Gracht E.T.I., Veerkamp D.M.B., Behr F.M., van Duikeren S., Beyrend G., Rip J., Nadafi R., Beyranvand Nejad E. (2022). A Third Vaccination with a Single T Cell Epitope Confers Protection in a Murine Model of SARS-CoV-2 Infection. Nat. Commun..

[168] Bilich T., Roerden M., Maringer Y., Nelde A., Heitmann J.S., Dubbelaar M.L., Peter A., Hörber S., Bauer J., Rieth J. (2021). Preexisting and Post-COVID-19 Immune Responses to SARS-CoV-2 in Patients with Cancer. Cancer Discov..

[169] Bange E.M., Han N.A., Wileyto P., Kim J.Y., Gouma S., Robinson J., Greenplate A.R., Hwee M.A., Porterfield F., Owoyemi O. (2021). CD8+ T Cells Contribute to Survival in Patients with COVID-19 and Hematologic Cancer. Nat. Med..

[170] Kundu R., Narean J.S., Wang L., Fenn J., Pillay T., Fernandez N.D., Conibear E., Koycheva A., Davies M., Tolosa-Wright M. (2022). Cross-Reactive Memory T Cells Associate with Protection against SARS-CoV-2 Infection in COVID-19 Contacts. Nat. Commun..

[171] Grifoni A., Weiskopf D., Ramirez S.I., Mateus J., Dan J.M., Moderbacher C.R., Rawlings S.A., Sutherland A., Premkumar L., Jadi R.S. (2020). Targets of T Cell Responses to SARS-CoV-2 Coronavirus in Humans with COVID-19 Disease and Unexposed Individuals. Cell.

[172] Phetsouphanh C., Darley D.R., Wilson D.B., Howe A., Munier C.M.L., Patel S.K., Juno J.A., Burrell L.M., Kent S.J., Dore G.J. (2022). Immunological Dysfunction Persists for 8 Months Following Initial Mild-to-Moderate SARS-CoV-2 Infection. Nat. Immunol..

[173] Focosi D., McConnell S., Casadevall A., Cappello E., Valdiserra G., Tuccori M. (2022). Monoclonal Antibody Therapies against SARS-CoV-2. Lancet Infect. Dis..

[174] Dagotto G., Ventura J.D., Martinez D.R., Anioke T., Chung B.S., Siamatu M., Barrett J., Miller J., Schäfer A., Yu J. (2022). Immunogenicity and Protective Efficacy of a Rhesus Adenoviral Vaccine Targeting Conserved COVID-19 Replication Transcription Complex. npj Vaccines.

[175] Thieme C.J., Anft M., Paniskaki K., Blazquez-Navarro A., Doevelaar A., Seibert F.S., Hoelzer B., Konik M.J., Berger M.M., Brenner T. (2020). Robust T Cell Response Toward Spike, Membrane, and Nucleocapsid SARS-CoV-2 Proteins Is Not Associated with Recovery in Critical COVID-19 Patients. Cell Rep. Med..

[176] Govender M., Hopkins F.R., Göransson R., Svanberg C., Shankar E.M., Hjorth M., Nilsdotter-Augustinsson Å., Sjöwall J., Nyström S., Larsson M. (2022). T Cell Perturbations Persist for at Least 6 Months Following Hospitalization for COVID-19. Front. Immunol..

[177] Flacco M.E., Acuti Martellucci C., Baccolini V., De Vito C., Renzi E., Villari P., Manzoli L. (2022). Risk of Reinfection and Disease after SARS-CoV-2 Primary Infection: Meta-Analysis. Eur. J. Clin. Investig..

[178] Bacher P., Rosati E., Esser D., Martini G.R., Saggau C., Schiminsky E., Dargvainiene J., Schröder I., Wieters I., Khodamoradi Y. (2020). Low-Avidity CD4+ T Cell Responses to SARS-CoV-2 in Unexposed Individuals and Humans with Severe COVID-19. Immunity.

[179] Murray S.M., Ansari A.M., Frater J., Klenerman P., Dunachie S., Barnes E., Ogbe A. (2023). The Impact of Pre-Existing Cross-Reactive Immunity on SARS-CoV-2 Infection and Vaccine Responses. Nat. Rev. Immunol..

[180] Forni D., Cagliani R., Pontremoli C., Mozzi A., Pozzoli U., Clerici M., Sironi M. (2020). Antigenic Variation of SARS-CoV-2 in Response to Immune Pressure. Mol. Ecol..

[181] Nelde A., Bilich T., Heitmann J.S., Maringer Y., Salih H.R., Roerden M., Lübke M., Bauer J., Rieth J., Wacker M. (2021). SARS-CoV-2-Derived Peptides Define Heterologous and COVID-19-Induced T Cell Recognition. Nat. Immunol..

[182] Le Bert N., Tan A.T., Kunasegaran K., Tham C.Y.L., Hafezi M., Chia A., Chng M.H.Y., Lin M., Tan N., Linster M. (2020). SARS-CoV-2-Specific T Cell Immunity in Cases of COVID-19 and SARS, and Uninfected Controls. Nature.

[183] Zahran A.M., Nafady-Hego H., Rashad A., El-Badawy O., Nasif K.A., Mostafa A.T., Osman H.A., Dongol E.M., Hashim A.A., Abdelrazek G.M. (2022). Increased Percentage of Apoptotic and CTLA-4 (CD152) Expressing Cells in CD4+/CD8+ Cells in COVID-19 Patients. Medicine.

[184] Chen G., Wu D., Guo W., Cao Y., Huang D., Wang H., Wang T., Zhang X., Chen H., Yu H. (2020). Clinical and Immunological Features of Severe and Moderate Coronavirus Disease 2019. J. Clin. Investig..

[185] Zheng M., Gao Y., Wang G., Song G., Liu S., Sun D., Xu Y., Tian Z. (2020). Functional Exhaustion of Antiviral Lymphocytes in COVID-19 Patients. Cell Mol. Immunol..

[186] Wang F., Nie J., Wang H., Zhao Q., Xiong Y., Deng L., Song S., Ma Z., Mo P., Zhang Y. (2020). Characteristics of Peripheral Lymphocyte Subset Alteration in COVID-19 Pneumonia. J. Infect. Dis..

[187] Arshad N., Laurent-Rolle M., Ahmed W.S., Hsu J.C.-C., Mitchell S.M., Pawlak J., Sengupta D., Biswas K.H., Cresswell P. (2023). SARS-CoV-2 Accessory Proteins ORF7a and ORF3a Use Distinct Mechanisms to down-Regulate MHC-I Surface Expression. Proc. Natl. Acad. Sci. USA.

[188] Zhang Y., Chen Y., Li Y., Huang F., Luo B., Yuan Y., Xia B., Ma X., Yang T., Yu F. (2021). The ORF8 Protein of SARS-CoV-2 Mediates Immune Evasion through down-Regulating MHC-Ι. Proc. Natl. Acad. Sci. USA.

[189] Maher A.K., Burnham K.L., Jones E.M., Tan M.M.H., Saputil R.C., Baillon L., Selck C., Giang N., Argüello R., Pillay C. (2022). Transcriptional Reprogramming from Innate Immune Functions to a Pro-Thrombotic Signature by Monocytes in COVID-19. Nat. Commun..

[190] Shen X.-R., Geng R., Li Q., Chen Y., Li S.-F., Wang Q., Min J., Yang Y., Li B., Jiang R.-D. (2022). ACE2-Independent Infection of T Lymphocytes by SARS-CoV-2. Signal Transduct. Target. Ther..

[191] Brunetti N.S., Davanzo G.G., de Moraes D., Ferrari A.J., Souza G.F., Muraro S.P., Knittel T.L., Boldrini V.O., Monteiro L.B., Virgílio-da-Silva J.V. (2023). SARS-CoV-2 uses CD4 to infect T helper lymphocytes. Elife.

[192] Lymphocytopenia–Hematology and Oncology. https://www.merckmanuals.com/professional/hematology-and-oncology/leukopenias/lymphocytopenia.

[193] Diao B., Wang C., Tan Y., Chen X., Liu Y., Ning L., Chen L., Li M., Liu Y., Wang G. (2020). Reduction and Functional Exhaustion of T Cells in Patients With Coronavirus Disease 2019 (COVID-19). Front. Immunol..

[194] Zheng H.-Y., Zhang M., Yang C.-X., Zhang N., Wang X.-C., Yang X.-P., Dong X.-Q., Zheng Y.-T. (2020). Elevated Exhaustion Levels and Reduced Functional Diversity of T Cells in Peripheral Blood May Predict Severe Progression in COVID-19 Patients. Cell Mol. Immunol..

[195] Cizmecioglu A., Akay Cizmecioglu H., Goktepe M.H., Emsen A., Korkmaz C., Esenkaya Tasbent F., Colkesen F., Artac H. (2021). Apoptosis-Induced T-Cell Lymphopenia Is Related to COVID-19 Severity. J. Med. Virol..

[196] André S., Picard M., Cezar R., Roux-Dalvai F., Alleaume-Butaux A., Soundaramourty C., Cruz A.S., Mendes-Frias A., Gotti C., Leclercq M. (2022). T Cell Apoptosis Characterizes Severe Covid-19 Disease. Cell Death Differ..

[197] Tan L., Wang Q., Zhang D., Ding J., Huang Q., Tang Y.-Q., Wang Q., Miao H. (2020). Lymphopenia Predicts Disease Severity of COVID-19: A Descriptive and Predictive Study. Signal Transduct. Target. Ther..

[198] Files J.K., Boppana S., Perez M.D., Sarkar S., Lowman K.E., Qin K., Sterrett S., Carlin E., Bansal A., Sabbaj S. (2021). Sustained Cellular Immune Dysregulation in Individuals Recovering from SARS-CoV-2 Infection. J. Clin. Investig..

[199] Danwang C., Noubiap J.J., Robert A., Yombi J.C. (2022). Outcomes of Patients with HIV and COVID-19 Co-Infection: A Systematic Review and Meta-Analysis. AIDS Res. Ther..

[200] McMahan K., Yu J., Mercado N.B., Loos C., Tostanoski L.H., Chandrashekar A., Liu J., Peter L., Atyeo C., Zhu A. (2021). Correlates of Protection against SARS-CoV-2 in Rhesus Macaques. Nature.

[201] Hasenkrug K.J., Feldmann F., Myers L., Santiago M.L., Guo K., Barrett B.S., Mickens K.L., Carmody A., Okumura A., Rao D. (2021). Recovery from Acute SARS-CoV-2 Infection and Development of Anamnestic Immune Responses in T Cell-Depleted Rhesus Macaques. mBio.

[202] Reynolds J., Shojania K., Marra C.A. (2007). Abatacept: A Novel Treatment for Moderate-to-Severe Rheumatoid Arthritis. Pharmacotherapy.

[203] Chitale S., Moots R. (2008). Abatacept: The First T Lymphocyte Co-Stimulation Modulator, for the Treatment of Rheumatoid Arthritis. Expert Opin. Biol. Ther..

[204] Vincenti F. (2008). Costimulation Blockade in Autoimmunity and Transplantation. J. Allergy Clin. Immunol..

[205] Ko E.R., Anstrom K.J., Panettieri R.A., Lachiewicz A.M., Maillo M., O’Halloran J.A., Boucher C., Smith P.B., McCarthy M.W., Segura Nunez P. (2022). Abatacept for Treatment of Adults Hospitalized with Moderate or Severe COVID-19. medRxiv.

[206] Bristol Myers Squibb Announces Topline Results Showing Treatment with Orencia (Abatacept) Improved Survival in People Hospitalized with COVID-19. https://news.bms.com/news/details/2022/Bristol-Myers-Squibb-Announces-Topline-Results-Showing-Treatment-with-Orencia-abatacept-Improved-Survival-in-People-Hospitalized-with-COVID-19/default.aspx.

[207] Hamdy A., Leonardi A. (2022). Superantigens and SARS-CoV-2. Pathogens.

[208] Schreibing F., Hannani M.T., Kim H., Nagai J.S., Ticconi F., Fewings E., Bleckwehl T., Begemann M., Torow N., Kuppe C. (2022). Dissecting CD8+ T Cell Pathology of Severe SARS-CoV-2 Infection by Single-Cell Immunoprofiling. Front. Immunol..

[209] Georg P., Astaburuaga-García R., Bonaguro L., Brumhard S., Michalick L., Lippert L.J., Kostevc T., Gäbel C., Schneider M., Streitz M. (2022). Complement Activation Induces Excessive T Cell Cytotoxicity in Severe COVID-19. Cell.

[210] Kalfaoglu B., Almeida-Santos J., Tye C.A., Satou Y., Ono M. (2021). T-Cell Dysregulation in COVID-19. Biochem. Biophys. Res. Commun..

[211] Zeng C., Evans J.P., Reisinger S., Woyach J., Liscynesky C., Boghdadly Z.E., Rubinstein M.P., Chakravarthy K., Saif L., Oltz E.M. (2021). Impaired Neutralizing Antibody Response to COVID-19 mRNA Vaccines in Cancer Patients. medRxiv.

[212] Terpos E., Stellas D., Rosati M., Sergentanis T.N., Hu X., Politou M., Pappa V., Ntanasis-Stathopoulos I., Karaliota S., Bear J. (2021). SARS-CoV-2 Antibody Kinetics Eight Months from COVID-19 Onset: Persistence of Spike Antibodies but Loss of Neutralizing Antibodies in 24% of Convalescent Plasma Donors. Eur. J. Intern. Med..

[213] Hartl D.L., Clark A.G. (1997). Principles of Population Genetics.

[214] Gillespie J.H. (1998). Population Genetics.

[215] Cao Y., Wang J., Jian F., Xiao T., Song W., Yisimayi A., Huang W., Li Q., Wang P., An R. (2022). Omicron Escapes the Majority of Existing SARS-CoV-2 Neutralizing Antibodies. Nature.

[216] I Think There’s Perhaps Been Some Confusion Regarding Transmissibility vs Immune Escape in Omicron. https://twitter.com/trvrb/status/1465364300936085506?lang=en.

[217] The Biden Administration Is Pushing New Covid Boosters. Who’s Listening?. https://www.statnews.com/2022/08/31/the-biden-administration-is-pushing-another-round-of-covid-boosters-whos-listening/.

[218] Rubin R. (2021). COVID-19 Vaccine Makers Plan for Annual Boosters, but It’s Not Clear They’ll Be Needed. JAMA.

[219] Scientists Said We’d Take Annual COVID Jabs like Flu Shots. Now Fauci Says It Might Be Only Every 5 Years. https://fortune.com/2022/02/09/scientists-said-wed-take-annual-covid-jabs-like-flu-shots-now-fauci-says-it-might-be-only-every-5-years/.

[220] KFF COVID-19 Vaccine Monitor: December 2022–Methodology. https://www.kff.org/report-section/kff-covid-19-vaccine-monitor-december-2022-methodology/.

[221] Canetti M., Barda N., Gilboa M., Indenbaum V., Asraf K., Gonen T., Weiss-Ottolenghi Y., Amit S., Doolman R., Mendelson E. (2022). Six-Month Follow-up after a Fourth BNT162b2 Vaccine Dose. N. Engl. J. Med..

[222] Bar-On Y.M., Goldberg Y., Mandel M., Bodenheimer O., Amir O., Freedman L., Alroy-Preis S., Ash N., Huppert A., Milo R. (2022). Protection by a Fourth Dose of BNT162b2 against Omicron in Israel. N. Engl. J. Med..

[223] Stay Up to Date with COVID-19 Vaccines. https://www.cdc.gov/covid/vaccines/stay-up-to-date.html.

