# Supplementary material for: The Impact of Vaccination Frequency on COVID-19 Public Health Outcomes: A Model-Based Analysis
Source: Vaccines (Basel). 2025 Mar 30;13(4):368. doi: 10.3390/vaccines13040368 (PMC12031506; doi:10.3390/vaccines13040368)
Supplement: Supplementary file 1 [file vaccines-13-00368-s001.zip › vaccines-3420804-supplementary.pdf]

The impact of vaccination frequency on COVID-19 public health outcomes: A model-based analysis

Authors: Madison Stoddard[1], Lin Yuan[1], Sharanya Sarkar[2], Debra van Egeren[3], Shruthi Mangalaganesh[4], Ryan P. Nolan[5], Michael S. Rogers[6,7], Greg Hather[8], Laura F. White[9], Arijit Chakravarty[1*]

Affiliations:
[1] Fractal Therapeutics, Cambridge, MA, USA.
[2] Dartmouth College, Hanover, NH, USA.
[3] Stanford University School of Medicine, Stanford, CA, USA.
[4] Monash University, Melbourne, VIC, AUS.
[5] Halozyme Therapeutics, San Diego, CA, USA.
[6] Harvard Medical School, Boston, MA, USA.
[7] Boston Children's Hospital, Boston, MA, USA.
[8] Sage Therapeutics, Cambridge, MA, USA.
[9] Boston University School of Public Health, Boston, MA, USA.

* Corresponding author
Email: arijit@fractal.tx (A.C.)

Abstract:

While the rapid deployment of SARS-CoV-2 vaccines had a significant impact on the ongoing COVID-19 pandemic, rapid viral immune evasion and waning neutralizing antibody titers have degraded vaccine efficacy. Nevertheless, vaccine manufacturers and public health authorities have a number of levers at their disposal to maximize the benefits of vaccination. Here, we use an agent-based modeling framework coupled with the outputs of a population pharmacokinetic model to examine the impact of boosting frequency and durability of vaccinal response on vaccine efficacy. Our work suggests that repeated dosing at frequent intervals (multiple times a year) may offset the degradation of vaccine efficacy, preserving their utility in managing the ongoing pandemic. Our work relies on assumptions about antibody accumulation and the tolerability of repeated vaccine doses. Given the practical significance of potential improvements in vaccinal utility, clinical research to better understand the effects of repeated vaccination would be highly impactful. These findings are particularly relevant as public health authorities worldwide seek to reduce the frequency of boosters to once a year or less. Our work suggests practical recommendations for vaccine manufacturers and public health authorities and draws attention to the possibility that better outcomes for SARS-CoV-2 public health remain within reach.

Supplementary file

Section S1: Lack of evidence for a protective role for T cell immunity for SARS-CoV-2

Summary

A precise understanding of the role of T cells in COVID-19 is critical for the design of biomedical interventions as well as public health strategy for the ongoing pandemic. Here we discuss the relative importance of T cells in protecting against infection by the SARS-CoV-2 virus and also against the risk of developing severe COVID-19. We will contrast this role against the role played by neutralizing antibodies (nAbs) produced as part of the B cell-mediated (humoral) adaptive immune response.

At this point, the weight of evidence argues against a role for T cells in providing protective immunity against SARS-CoV-2. Early in the pandemic, a body of studies demonstrated convincingly that the T cell response is durable after both infection and vaccination (See **Footnote A** below for details). These encouraging findings led to the widely held perception that sustained immunity against severe disease [1–3] and protection against viral immune evasion [4, 5] would be provided by the observed durability of T cell responses. This hope was widely expressed in the public sphere as well [1, 2, 6, 7]. However, vaccinal immunity against severe disease and death (VE_s) has been observed to wane rapidly over time and in response to viral evolution (See **Footnote B** below). This rapidly waning VE_s, despite the presence of a durable T cell response, creates a logical contradiction in the proposition that T cells play a role in providing protective immunity.

In addition, while certain pools of T cells (in particular SARS-CoV-2-specific CD8+ and CD4+ T cells) are frequently found in convalescent patients, overall T cell numbers decrease during infection with SARS-CoV-2, in both mild and severe cases (See **Footnote C**), with T cells being infected directly by the virus and undergoing apoptosis as a result (**Footnote D**). Conflating the post-infection increase in the relative frequencies of CD4+ and CD8+ T cells with their absolute numbers is a source of confusion on this point. Further, the loss of T cells (either due to disease or drug treatment) does not result in worse outcomes for COVID-19 disease progression (See **Footnote E**), and the T cell blockading drug Abatacept has shown a positive impact on disease outcome in COVID-19 patients in a clinical setting (**Footnote E**), suggesting a net negative impact of T-cell response on disease pathophysiology. Notably, SARS-CoV-2-specific CD8+ and CD4+ T cells have also been found in unexposed controls from the beginning of the pandemic; these are thought to arise as a result of cross-reactivity with common cold coronaviruses (**Footnote C**). Despite reports to the contrary, T cells levels are not predictive of protection against SARS-

CoV-2 infection (**Footnote F**). Similarly, reports of T cell-mediated protection in the absence of a functional B cell (or nAb) response have been prone to issues with interpretation of the underlying data (**Footnote G**).

On the other hand, a strong case can be made that protection against SARS-CoV-2 infection is provided by nAbs. nAbs are a validated correlate of immune protection, and they have been shown in multiple studies to be predictive of vaccinal protection for both symptomatic infections and severe disease, across a range of vaccines and viral variants (See **Footnote H** for details). Drugs and conditions that impair B cell function or lead to lower nAb levels are strongly associated with worse outcomes for SARS-CoV-2 infection (see **Footnote I**). Restoration of nAb levels is strongly associated with improved outcomes (See **Footnote J**). Limited preclinical data suggests that this is not the case for T cell functionality for SARS-CoV-2 (**Footnote J**).

The observed evolutionary trajectory of the SARS-CoV-2 virus adds further support for the argument that humoral and not cellular immunity is the primary source of protection for COVID-19. It is a basic principle in evolutionary biology that the strength of the selection pressure determines the rate of evolution of resistance [8, 9]. Currently, viral evolution is being primarily driven by immune evasion-specifically against nAbs [10, 11]. We predicted this rapid evolution of immune evasion using an evolutionary biology framework in a paper [12] whose preprint was posted in the fall of 2020 [13]. That work started from the premise that neutralizing antibodies would exert a selection pressure that the virus would need to evade, and we demonstrated that rapid immune evasion could be possible under conditions where there was a large standing pool of SARS-CoV-2 infections. Thus, rapid evolution of immune evasion to nAbs validates their impact on viral fitness. Conversely, the evolutionary stability of the T cell epitopes is consistent with no selection pressure being exerted by the T cells on SARS-CoV-2. (The alternative explanation for the evolutionary stability of the T cell epitopes, that they cannot be evaded by the virus, has been undermined by the rapid reductions in VE_s due to evolutionary immune evasion).

In summary, multiple lines of evidence argue against a role for T cells in protection against SARS-CoV-2 infection. The observed durability and evolutionary stability of the T cell response has not translated into lasting protection against infection or severe disease.

Footnotes:

A: T cell response is persistent and evolutionarily stable

T cell response is durable after infection [14–17]. The fraction of spike-specific T cells goes up rapidly upon vaccination [18], and the T cell response in vaccinated individuals is also persistent [1, 2, 14]. T cell (but not B cell) epitopes are evolutionarily stable- while B cell epitopes in the spike glycoprotein (S) and in the nucleocapsid protein (N) have higher diversity than nonepitope positions, epitopes for CD4+ and CD8+ T cells are not more variable than nonepitope positions [19]. The T cell response remains durable even in the face of the newer immune-evading variants [5, 20–24] and the vaccinal and natural T cell responses to Omicron BA.1, for example, are broadly preserved [25–27].

While a number of papers have reported immune escape mutations at T cell epitopes, we note that many of these mutations are not present in the currently circulating SARS-CoV-2 lineages (Table 1)

Protein	Substitution	T cell class	Current frequency	Reference
S	N969K	CD4+	93%	[28]
S	P272L	CD8+	0%	[29]
S	A1022S	CD4+	0%	[30]
S	T1072I	CD4+	0%	[30]
S	E484K	CD8+	0%	[31]
N	T362I	CD8+	0%	[32]
N	P365S	CD8+	0%	[32]
N	P13L	CD8+	93%	[32]
ORF3a	Q213K	CD8+	0%	[32]
ORF1a	T147I	CD8+	0%	[33]
N	P344L	CD8+	0%	[33]
M	A69V	CD8+	0%	[33]
ORF1a	I2230T	CD8+	0%	[34]
S	L452R	CD8+	73%	[34]
S	L822P	CD8+	0%	[33]

Table S1: Reported immune escape mutations in the T cell epitopes of the spike protein, with their current frequency in the cov-spectrum database (as of 01/19/23) [35]. List is not exhaustive, see [36] for additional examples of reported T cell-evasive mutations.

B: Vaccinal efficacy against severe disease (VE_s) is limited, and vulnerable to immune evasion.

)A recent CDC study showed that the vaccine effectiveness (a measure of VE_i) of a bivalent mRNA COVID-19 booster received after 2 or more doses of monovalent vaccines ranged from 43% (for the 18-49 age group) to 22% (for the over-65 age group) [37]. In the original placebo controlled trials among mostly naïve individuals, absolute vaccine efficacy (VE_s) was originally observed to be very high [38, 39], however over time this efficacy waned in concert with nAb titers in both placebo-controlled trials [40] and large-scale observational studies before the omicron wave dramatically increased the background rate of immunity [41–45]. More recent trials in much more extensively vaccinated and infected populations confirm declines in VE_s over time that can be increased by boosting [46]. When it comes to severe acute disease, VE_s for a newly boosted individual is now 56% [40]. Continued viral evolution has been degrading VE_s [47–50], although it is partially restored with boosters [51]. Two-dose vaccinal series have only a modest benefit (VE_s~50%) against severe disease with Omicron [47, 52–54] despite these vaccinations having been demonstrated to elicit a T cell response that is both persistent and conserved across variants of concern [21–23, 52].

C: Issues with interpretation of T cell levels

Both unexposed individuals and those convalescing from severe COVID-19 have SARS-CoV-2-reactive T cells [55–57], particularly CD4+ and CD8+. Some proportion of these SARS-CoV-2 reactive T cell populations are cross-reactive with common cold coronaviruses [56, 58–60]. While SARS-CoV-2-specific CD8+ and CD4+ T cells are more commonly found in convalescent patients than in unexposed controls, overall T cell levels are not elevated in convalescent patients [56]. Notably, the absolute counts of total lymphocytes and CD4+ and CD8+ T cells in hospitalized as well as non-hospitalized COVID cases are lower than those in healthy counterparts ([61], see Table 1 and 2; [62], see Table 3; [63], see Figure 1; [64], see Figures 1 &2).

D: SARS-CoV-2 infection actively suppresses the T cell response.

SARS-CoV-2 inhibits the induction of the MHC-I pathway in cells infected by the virus (thereby evading T cell recognition) by several different molecular mechanisms [33, 65, 66]. Viral infections also impair the capacity of monocytes to activate SARS-CoV-2 CD4+ and CD8+ T cells [67]. In addition to impairing T cell activation, SARS-CoV-2 also damages and depletes T cells directly [68]. T cells are directly infected by SARS-CoV-2 [69, 70], express markers of functional exhaustion following severe COVID [71, 72] and

undergo frank apoptosis during viral infection [71, 73, 74]. Notably, T cell lymphopenia and dysregulation are observed in both mild (non-hospitalized) and severe cases of COVID [61, 71–77].

E. Loss of T cell function does not negatively impact outcomes upon SARS-CoV-2 infection.

T cell depletion via disease or therapeutic intervention has not directly been demonstrated to lead to worse outcomes for SARS-CoV-2 infection. A meta-analysis of outcomes of patients with HIV and SARS-CoV-2 coinfection showed that HIV was not associated with the severity of the disease (OR: 1.28; 95% CI 0.77-2.13, 13 studies), or death (OR: 0.81; 95% CI 0.47; 1.41, 23 studies) in COVID-19 patients, although HIV was independently associated with an increased risk of hospitalization (OR: 1.49; 95% CI 1.01-2.21, 6 studies) and death (hazard ratio: 1.76, 95% CI 1.31-2.35, 2 studies) compared to HIV-negative individuals [78]. Direct depletion of T cells similarly has no impact on COVID-19 outcomes. T cell depletion in macaques does not lead to higher viral loads in the lung [79], nor does it disrupt protection against short-term reinfection [80]. Similar findings have been reported with the ablation of T cell function in humans. Abatacept, used for treatment of rheumatoid arthritis (RA), blocks T cell co-stimulation and decreases T cell activation [81–83]. Abatacept treatment is not associated with worse outcomes for RA patients infected with SARS-CoV-2 (HR: 1.14, 95% C.I.: 0.77 to 1.68), while B cell (humoral immunity)-depleting therapies lead to significantly worse outcomes in the same population: Rituximab (HR: 4.25, 95% C.I.: 3.17 to 5.69) and Janus Kinase inhibitors (HR: 2.05, 95% C.I.: 1.57 to 2.69) [84]. In fact, Abatacept treatment improves outcomes for COVID-19 [85, 86], consistent with the negative role for T cells in the disease that has been proposed by a number of researchers [87–90].

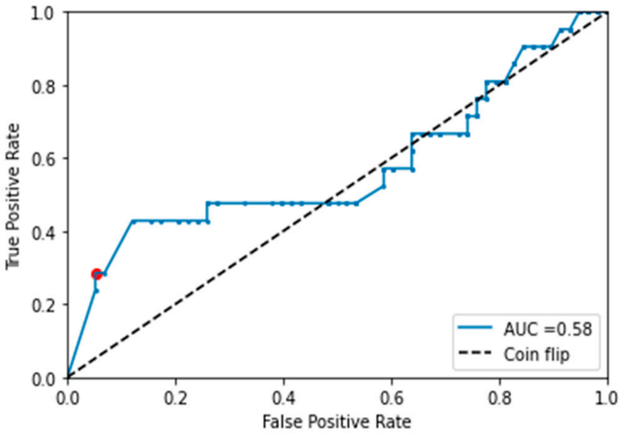

Figure S1: ROC curve describing spike-specific T cell frequency as a predictor of testing positive for SARS-CoV-2. Red point represents threshold selected in the study.

F. T cells are not predictive of protection against SARS-CoV-2 infection.

The presence of SARS-CoV-2- specific T cells does not predict for protection against SARS-CoV-2 infection. We analyzed the dataset from Dowell, et al. [91] (examining the frequency of spike-specific T cells and subsequent positive SARS-CoV-2 test results in children) using a receiver operating characteristic (ROC) analysis [Fig S1]. Although the authors suggest an association between the presence of T cells

and protection against infection, we found that this association is weak. In this study, the authors measured spike-specific T cells and anti-spike IgG titers in enrollee children. The children's health records were monitored for later confirmed COVID-19. The authors selected thresholds for cellular and antibody positivity and assessed these as predictors of later test positivity. However, threshold choice can be expected to impact the apparent predictiveness of cellular and antibody positivity [92]. To assess the general predictive value of spike-specific T cell frequency, we constructed an ROC curve using the data in the study. We found that the area under the ROC curve (ROC AUC) is low at 0.58. For reference, a purely random predictor (i.e., a coin flip) will have an ROC AUC of 0.5, while a perfect predictor will achieve an ROC AUC of 1.

G. Studies reporting T cell protection in the absence of nAbs are prone to interpretation challenges. Both antibody and T cell responses are induced by primary SARS-CoV-2 infection in unvaccinated individuals, and the strength of the response across both arms of the immune system is correlated [56]. This makes it challenging to interpret studies attributing outcomes specifically to T cell function. For example, a recent study claimed that vaccines eliciting a stronger T cell response provided protection against SARS-CoV-2 infection in a murine model [93]. However, the vaccines that elicited higher levels of CD4+ T cells also elicited higher levels of nAb response, complicating the attribution of the improved protection to CD4+ T cells (See fig 1A and B in [93]).

Similar challenges have been associated with clinical studies- a number of studies have attempted to attribute the increased severity of COVID-19 in cancer patients, for example, to impaired T cell functionality [94, 95]; however, these patients often have an impaired nAb response as well [95–97]. A recent study identified a subset of patients with defective humoral immunity whose survival they attributed to CD8+ T cell counts ([95], see figure 4H). However, in this high-dimensional dataset, they selected a threshold for CD8 counts using a Classification and Regression Tree (CART) analysis, without specifying the use of a separate test and training set. The lack of cross-validation for a selected threshold makes the result challenging to interpret (as discussed in **Footnote F** above)- the result obtained here had a p-value of 0.04, which was not corrected for multiple comparisons. The lack of multiple comparisons corrections for statistical analysis on high-dimensional datasets is seen in other papers that claim an association between T cells and protection from infection more generally. For example, a recent paper [58] claimed that cross-reactive T cells protect against SARS-CoV-2 infection. The argument put forth by the authors hinges on a difference in frequency of IL2-secreting (but not IFN-γ or dual-

secreting) cross-reactive T cells, with an uncorrected t-test p-value of 0.0139. (See Figure 2. Notably, this was one of 12 comparisons shown in the figure!).

In other cases, study conclusions directly differ from the observed data. For example, one study claiming a role for CD8+ T cells in protection against SARS-CoV-2 showed that CD8+ immunodepleted macaques had no detectable viral load in the lungs upon viral rechallenge (similar to control, [79], see fig 4).

H. nAbs are a correlate of immunity

Neutralizing antibody (nAb) titers are a validated correlate of immune protection [53, 98–104] for SARS-CoV-2, as they are for other viruses [105]. nAb titers normalized to mean convalescent titer (from the same study) have been shown to fit well to a nonlinear dose-response relationship that is predictive of reported vaccinal protection across a range of different vaccines [101]. Two such dose-response curves exist, one linking nAb titers to protection against symptomatic infection, and one linking nAb titers to protection against severe COVID-19 outcomes. These relationships have held up across a range of studies [102, 103], and are also predictive of vaccinal efficacy against newly emerging variants [51, 104, 106–108]. Neutralizing antibody titers correlate with waning vaccinal protection against infection (VEi) due to PK waning [102, 109] or viral immune evasion [51, 104, 106–108] and with loss of vaccinal efficacy against severe disease (VEs) [101].

I. Loss of B cells or nAbs leads to dramatically worse outcomes upon SARS-CoV-2 infection

Rituximab suppresses the B cell, but not the T cell response for COVID-19 [110], and has been reported to lead to an increased risk of severe disease and/or death for COVID-19 patients in several studies [84, 111–115]. Similarly, CD20 therapy for multiple sclerosis, which suppresses humoral, but not cellular responses [116, 117], has been associated with an elevated risk of severe disease or death due to COVID-19 [115, 118]. Of note, these findings extend to vaccinal protection as well. Immunosuppressed patients have an impaired humoral, but not cellular response to COVID-19 vaccination [119]. Breakthrough infections for immunosuppressed patients have poor outcomes [120, 121], and VE_s in these populations is lower than that of the immunocompetent population [122–124].

J. Restoration of B cell (antibody), but not T cell function, mitigates COVID-19 severity

Prior to the evolution of viral resistance, monoclonal antibodies had a strongly beneficial impact on COVID-19 disease outcomes [125]. Conversely, B cell depleting therapies lead to worse outcomes for

breakthrough infections [120], which during the early phase of the pandemic could also be rescued by the use of monoclonal antibody therapies [120]. Vaccines that specifically generate a T cell response have minimal impact on COVID-19 in mouse models, while those that generate a B cell response do [126]. Robust T cell responses are not associated with recovery in critical COVID-19 patients [127]. Crucially, T cell levels take months to return to normal ranges after severe COVID [56, 128], which is inconsistent with the strong protection against reinfection typically observed immediately after recovery from acute COVID [129, 130]. (A subset of patients with mild-to-moderate SARS-CoV-2 infections also experience T cell depletion (including the loss of naïve T cells) and dysregulation [77].

Supplementary Figures

A.

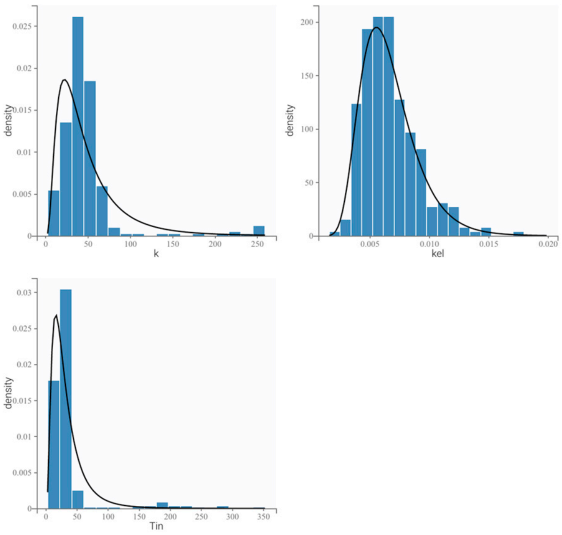

B.

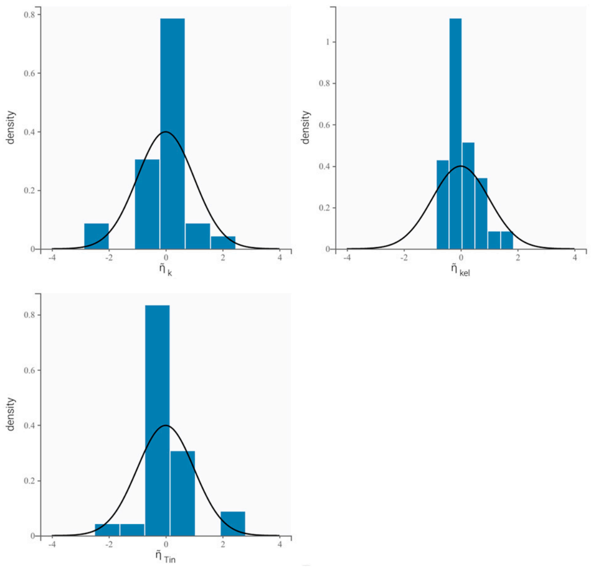

C.

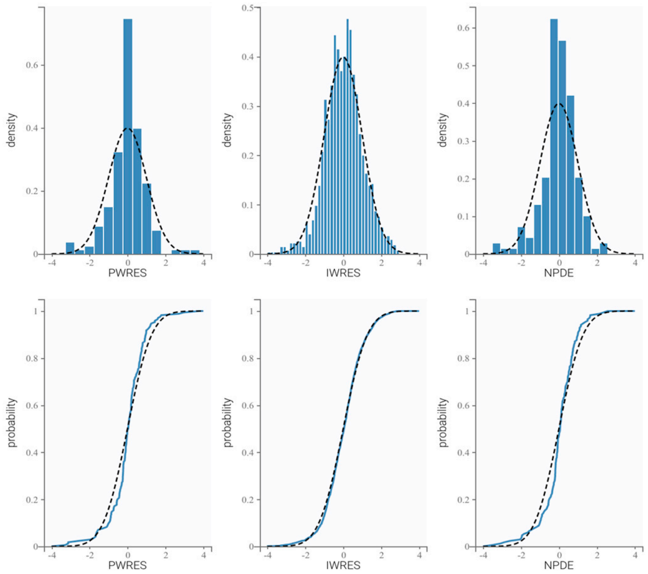
density
PWRES
IWRES
NPDE
probability

D.

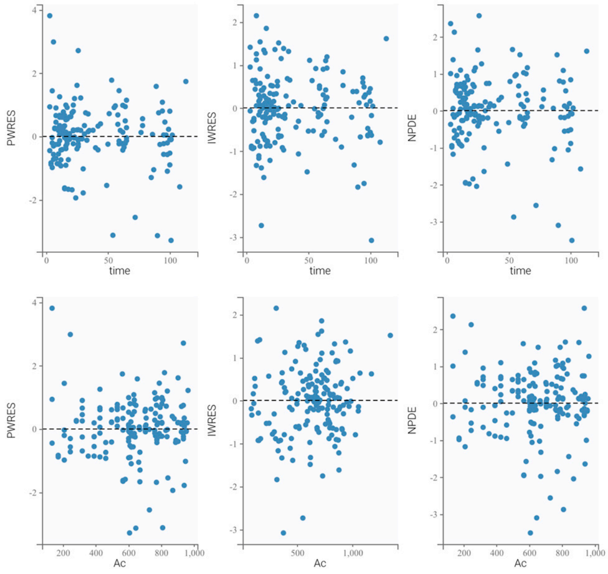
PWRES
IWRES
NPDE
time
Ac

E.

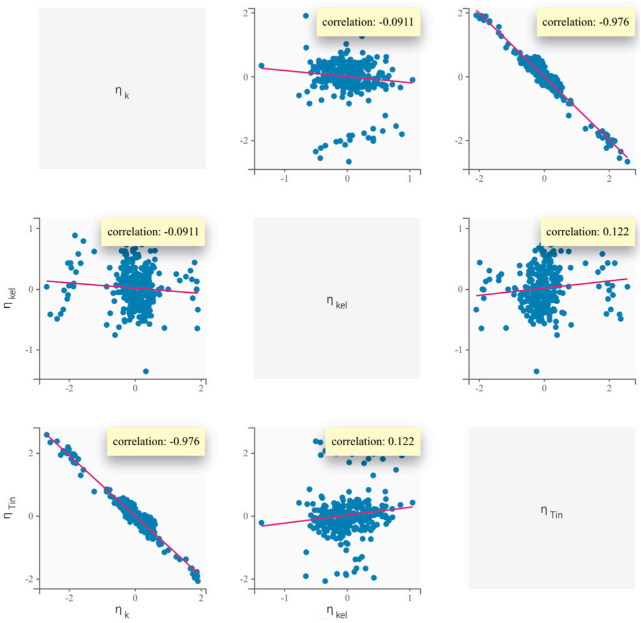

Figure S2. Goodness-of-fit assessment for nAb kinetics model describing antibody decay post-COVID-19 infection. **A.** Probability distribution of individual parameters. The histogram plots are empirical distributions. The black line shows the theoretical distribution defined in the statistical model, which is a log-normal distribution. **B.** Probability distribution of standardized random effects. The histogram plots are empirical distributions. The theoretical distribution is shown as a dotted curve which is a normal distribution. **C.** Scatter plots of the residuals. These plots display the PWRES (population weighted residuals), the IWRES (individual weighted residuals), and the NPDEs (normalized prediction distribution errors) as scatter plots with respect to the time and the prediction. residuals should be randomly scattered around the horizontal zero-line, which means the selected proportional error model is true. **D.** Distribution of the residuals. Empirical and theoretical probability density functions (PDF) of the PWRES, IWRES and NPDE are on top. Empirical and theoretical cumulative distribution functions (CDF) are at the bottom. Normally distributed residuals indicate that the selected error model is true. **E.** Correlations plots between model kinetic parameters. Solid lines represent the mean outcome while dashed lines delineate the 90% population interval.

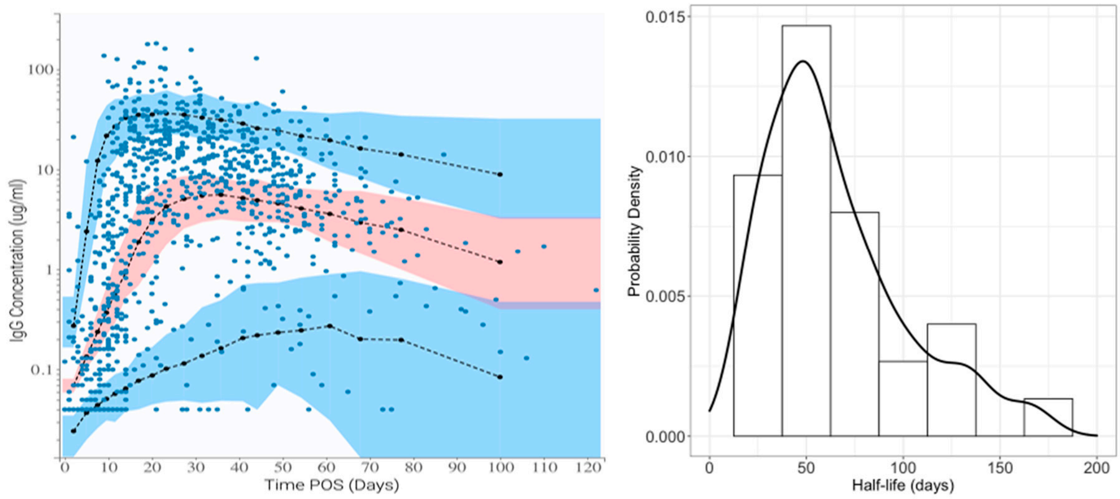

Figure S3. Mixed-effects model fit to IgG kinetics after SARS-CoV-2 infection. **A.** Visual predictive check for model agreement with fitted dataset. Shaded regions represent 90% prediction intervals for model certainty (pink area for the 50th percentile, and blue areas for the 10th and 90th percentiles). Blue dots represent the fitted dataset, with dark blue lines representing the empirical 10th and 90th percentiles and red lines representing the empirical 50th percentile. Distribution of **B**. IgG half-life probability density function.

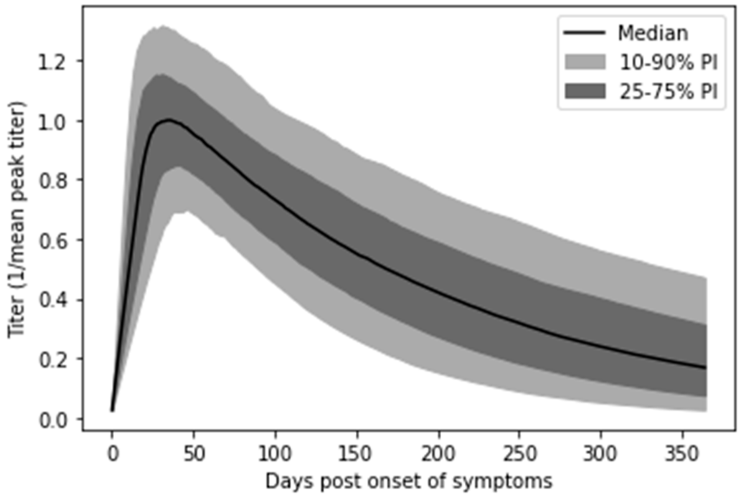

Figure S4. Normalized nAb titers post-onset of COVID-19 symptoms (POS) by percentile, as derived from simulations generated by the fitted model. After SARS-CoV-2 infection, neutralizing antibody titers wane across the population. Figure shows simulated antibody titers by percentile over time. Titers are normalized to the peak mean convalescent level, which is the mean titer at 34 days after onset of symptoms. A wide range of titers are observed at all timepoints, indicating significant variation in neutralizing antibody kinetics. In the 50th percentile, titers wane by approximately 75% within one year of symptom onset. By this point, neutralizing antibody titers have waned by more than 10-fold in the 10th percentile, while titers remain at about 60% of the mean peak convalescent level in the 90th percentile. These results suggest wide variation in peak neutralizing antibody titers and waning kinetics.

A. **B.**

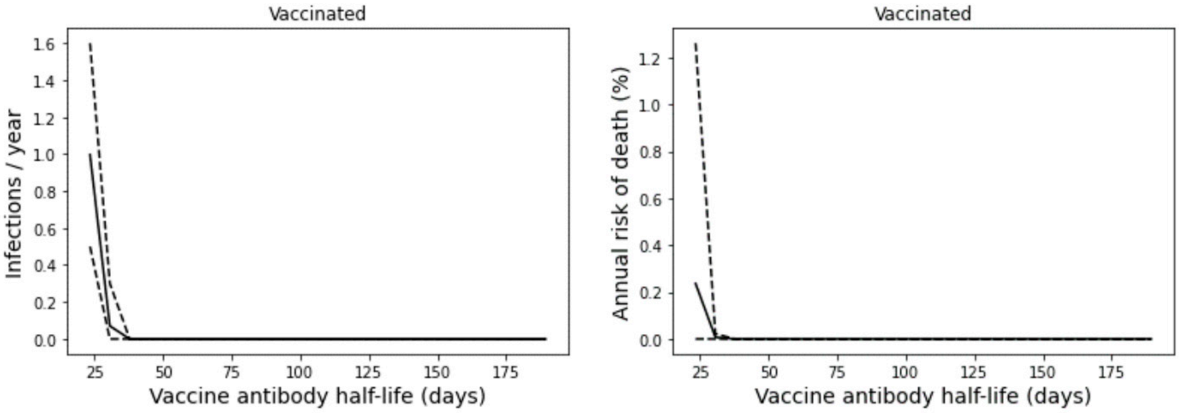

Figure S5: A more frequent vaccination schedule using current vaccines may benefit individuals with the shortest vaccine nAb half-lives. In this example, a five times yearly booster schedule is shown to prevent infections in all individuals with at least 40-day vaccine nAb half-lives. A six times yearly schedule was found to prevent infections in all immunocompetent individuals above the first percentile for vaccine nAb half-life. **A.** Average infection frequency and **B.** average yearly risk of death over a 10-year simulation. Dashed lines represent the 90% population interval.

A. B.

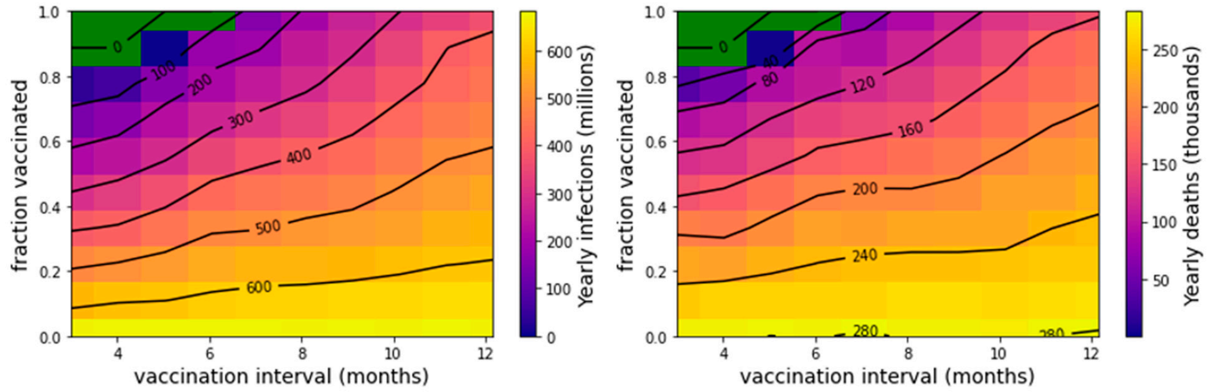

Figure S6. Increased durability of vaccine nAb titers improves population-level COVID-19 outcomes. **A.** Yearly US SARS-CoV-2 infections and **B.** deaths are simulated under a variety of vaccination frequency and compliance conditions. Green region represents complete suppression of SARS-CoV-2 transmission.

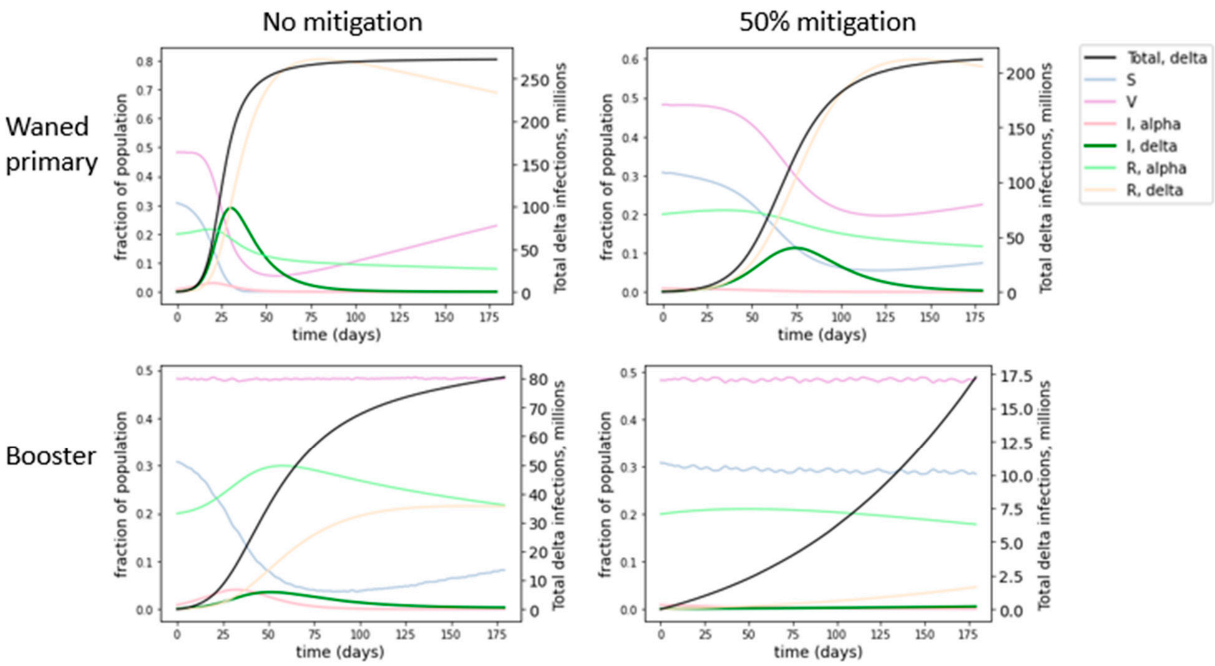

Figure S7: Boosters in the summer of 2021 could have blunted the impact of the delta wave in the US, even if greater adult compliance could not be achieved. In this figure series, we relax the assumption of perfect compliance in Figure 6, and instead assume that only 48% of the population is boosted; this is the fraction of the US population that had received the primary series at the time [110]. Each compartment in the SIRS model is represented by the fraction of individuals in the population in that compartment over time (left axis). S represents susceptible, V represents vaccinated, I represents infected with alpha or delta, R represents recovered from alpha or delta. The total number of delta infections is tracked (black line, right axis). These results suggest that a timely rollout of boosters in the summer of 2021 could have substantially blunted the delta wave (compare the green lines in the top and bottom panels), even in the absence of any other mitigation measures.

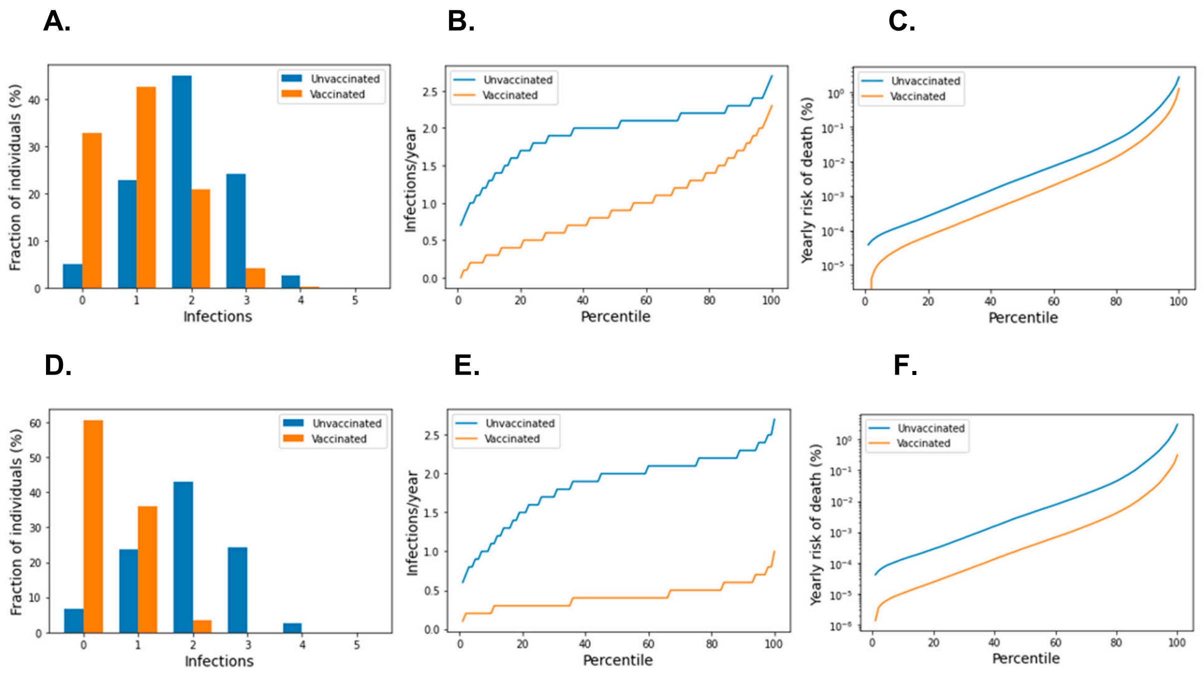

Figure S8. Impact of twice-yearly boosters on COVID-19 outcomes. Impact of simulated Moderna boosters on **A)** distribution of total infections in a given year, **B)** average yearly frequency of infection and **C)** average yearly risk of COVID-19 death. Impact of boosters with post-infection-like nAb kinetics on **D)** distribution of total infections in a given year, **E)** average yearly frequency of infection and **F)** average yearly risk of COVID-19 death.

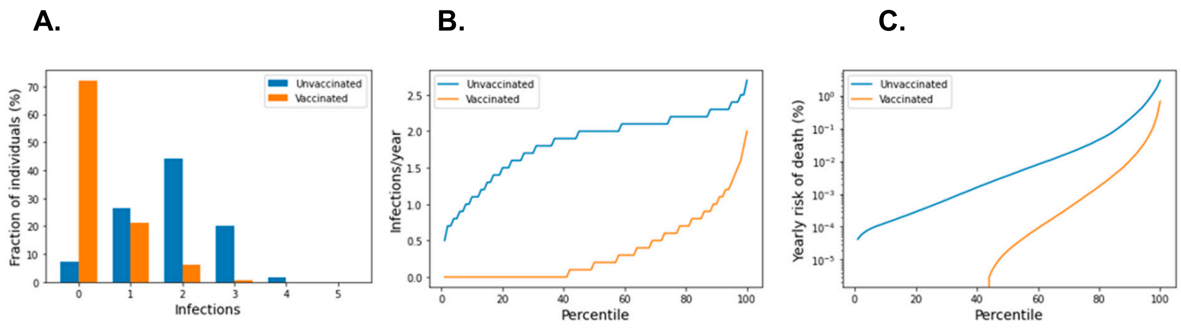

Figure S9. Impact of thrice-yearly Moderna boosters on COVID-19 outcomes. Impact of simulated Moderna boosters on **A)** distribution of total infections in a given year, **B)** average yearly frequency of infection and **C)** average yearly risk of COVID-19 death.

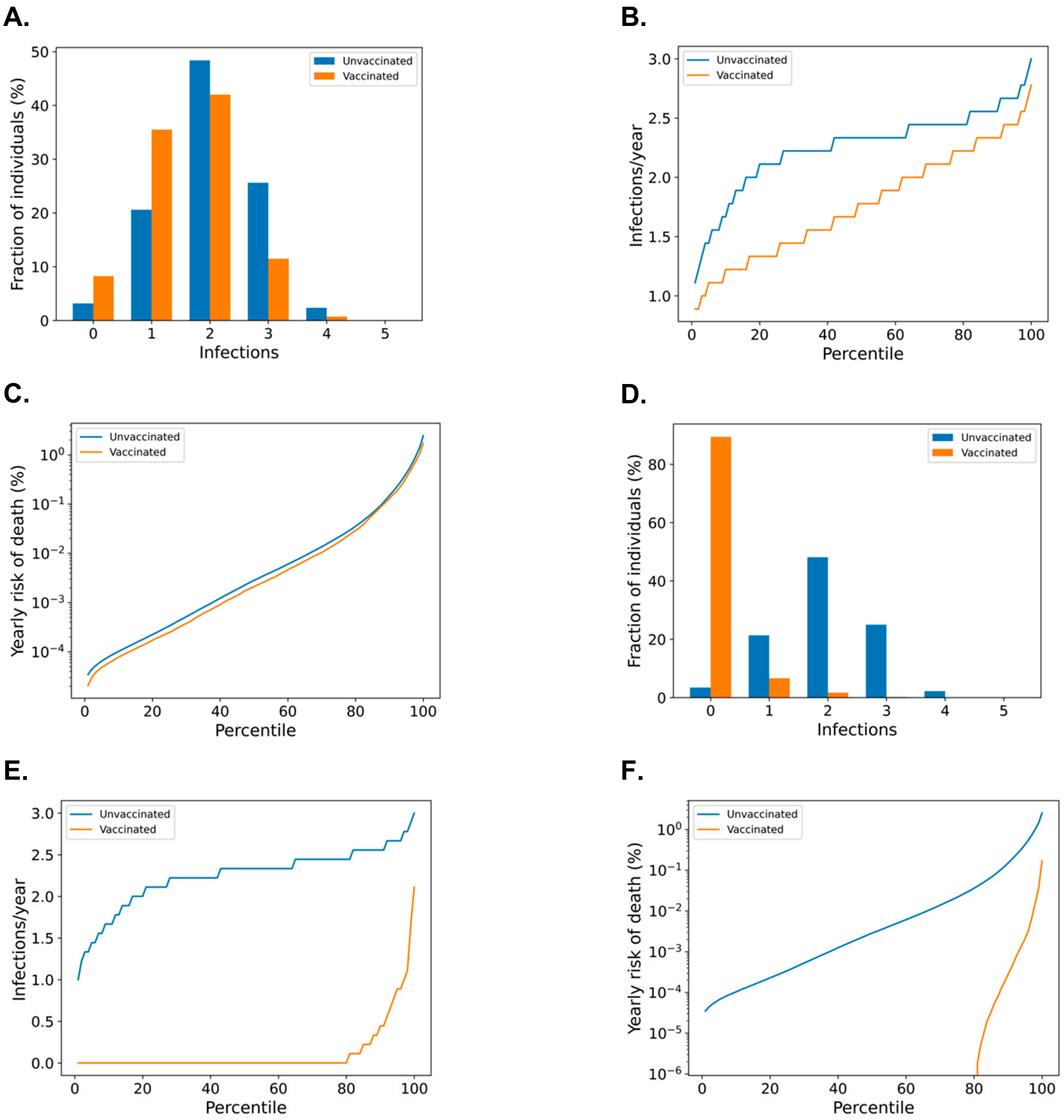

Figure S10. Impact of Moderna boosters on individual COVID-19 outcomes in the event of negligible booster uptake. For a once-yearly booster frequency, **A.** the distribution of infection counts in a single year, **B.** distribution of individual infection frequencies over a 10-year simulation, **and C**. interindividual heterogeneity in yearly risk of COVID-19 death. For a four-times yearly booster frequency, **D.** the distribution of infection counts in a single year, **E.** distribution of individual infection frequencies over a 10-year simulation, and **F.** interindividual heterogeneity in yearly risk of COVID-19 death. On a short-term basis (A. and D.), variation in infection risk is driven by interindividual heterogeneity in biology and behavior as well as stochasticity. In the long-term (B., C., E., and F.), interindividual heterogeneity dominates stochasticity in driving individual infection frequency and severe disease risk.

References

1. Board TE. Opinion | The T-Cell Covid Cavalry. WSJ. https://www.wsj.com/articles/the-t-cell-covid-cavalry-antibodies-vaccines-omicron-11640906490. Accessed 7 Jan 2023.

2. Leslie M. T cells found in coronavirus patients “bode well” for long-term immunity. Science. 2020;368:809–10.

3. T cells protect against COVID-19 in absence of antibody response. National Institutes of Health (NIH). 2022. https://www.nih.gov/news-events/nih-research-matters/t-cells-protect-against-covid-19-absence-antibody-response. Accessed 7 Jan 2023.

4. Grifoni A, Sidney J, Vita R, Peters B, Crotty S, Weiskopf D, et al. SARS-CoV-2 human T cell epitopes: Adaptive immune response against COVID-19. Cell Host Microbe. 2021;29:1076–92.

5. Tarke A, Sidney J, Methot N, Yu ED, Zhang Y, Dan JM, et al. Impact of SARS-CoV-2 variants on the total CD4+ and CD8+ T cell reactivity in infected or vaccinated individuals. Cell Rep Med. 2021;2:100355.

6. Opinion | Call in the T-Cell Cavalry to Fight COVID in the Immunocompromised. 2021. https://www.medpagetoday.com/opinion/second-opinions/93805. Accessed 7 Jan 2023.

7. Mannix L. The mystery that could explain why COVID vaccines work so well. The Sydney Morning Herald. 2021. https://www.smh.com.au/national/the-mystery-that-could-explain-why-covid-vaccines-work-so-well-20210427-p57mq0.html. Accessed 7 Jan 2023.

8. Hartl DL, Clark AG. Principles of population genetics. Sinauer Associates: Sunderland, MA; 1997.

9. Gillespie JH. Population genetics: a concise guide. Johns Hopkins University Press: Baltimore, MD; 1998.

10. Cao Y, Wang J, Jian F, Xiao T, Song W, Yisimayi A, et al. Omicron escapes the majority of existing SARS-CoV-2 neutralizing antibodies. Nature. 2022;602:657–63.

11. Trevor Bedford [@trvrb]. I think there’s perhaps been some confusion regarding transmissibility vs immune escape in Omicron. The apparent rapid increase in frequency of Omicron in Gauteng does not mean that Omicron is necessarily more intrinsically transmissible than Delta. 1/15. Twitter. 2021. https://twitter.com/trvrb/status/1465364300936085506. Accessed 7 Jan 2023.

12. Van Egeren D, Novokhodko A, Stoddard M, Tran U, Zetter B, Rogers M, et al. Risk of rapid evolutionary escape from biomedical interventions targeting SARS-CoV-2 spike protein. PLoS One. 2021;16:e0250780.

13. Egeren DV, Novokhodko A, Stoddard M, Tran U, Zetter B, Rogers M, et al. Risk of evolutionary escape from neutralizing antibodies targeting SARS-CoV-2 spike protein. 2020;:2020.11.17.20233726.

14. Guo L, Wang G, Wang Y, Zhang Q, Ren L, Gu X, et al. SARS-CoV-2-specific antibody and T-cell responses 1 year after infection in people recovered from COVID-19: a longitudinal cohort study. The Lancet Microbe. 2022;3:e348–56.

15. Immunological memory to SARS-CoV-2 assessed for up to 8 months after infection | Science. https://www.science.org/doi/10.1126/science.abf4063. Accessed 7 Jan 2023.

16. Cohen KW, Linderman SL, Moodie Z, Czartoski J, Lai L, Mantus G, et al. Longitudinal analysis shows durable and broad immune memory after SARS-CoV-2 infection with persisting antibody responses and memory B and T cells. Cell Rep Med. 2021;2:100354.

17. Tarke A, Coelho CH, Zhang Z, Dan JM, Yu ED, Methot N, et al. SARS-CoV-2 vaccination induces immunological T cell memory able to cross-recognize variants from Alpha to Omicron. Cell. 2022;185:847-859.e11.

18. Oberhardt V, Luxenburger H, Kemming J, Schulien I, Ciminski K, Giese S, et al. Rapid and stable mobilization of CD8+ T cells by SARS-CoV-2 mRNA vaccine. Nature. 2021;597:268–73.

19. Forni D, Cagliani R, Pontremoli C, Mozzi A, Pozzoli U, Clerici M, et al. Antigenic variation of SARS-CoV-2 in response to immune pressure. Molecular Ecology. 2021;30:3548–59.

20. Jing L, Wu X, Krist MP, Hsiang T-Y, Campbell VL, McClurkan CL, et al. T cell response to intact SARS-CoV-2 includes coronavirus cross-reactive and variant-specific components. JCI Insight. 2022;7:e158126.

21. Moss P. The T cell immune response against SARS-CoV-2. Nat Immunol. 2022;23:186–93.

22. Goel RR, Painter MM, Apostolidis SA, Mathew D, Meng W, Rosenfeld AM, et al. mRNA Vaccines Induce Durable Immune Memory to SARS-CoV-2 and Variants of Concern. Science. 2021;374:abm0829.

23. Low-dose mRNA-1273 COVID-19 vaccine generates durable memory enhanced by cross-reactive T cells | Science. https://www.science.org/doi/10.1126/science.abj9853. Accessed 7 Jan 2023.

24. Riou C, Keeton R, Moyo-Gwete T, Hermanus T, Kgagudi P, Baguma R, et al. Escape from recognition of SARS-CoV-2 variant spike epitopes but overall preservation of T cell immunity. Science Translational Medicine. 2021;14:eabj6824.

25. May DH, Rubin BER, Dalai SC, Patel K, Shafiani S, Elyanow R, et al. Immunosequencing and epitope mapping reveal substantial preservation of the T cell immune response to Omicron generated by SARS-CoV-2 vaccines. 2021;:2021.12.20.21267877.

26. Ahmed SF, Quadeer AA, McKay MR. SARS-CoV-2 T Cell Responses Elicited by COVID-19 Vaccines or Infection Are Expected to Remain Robust against Omicron. Viruses. 2022;14:79.

27. De Marco L, D'Orso S, Pirronello M, Verdiani A, Termine A, Fabrizio C, et al. Assessment of T-cell Reactivity to the SARS-CoV-2 Omicron Variant by Immunized Individuals. JAMA Network Open. 2022;5:e2210871.

28. Pastorio C, Zech F, Noettger S, Jung C, Jacob T, Sanderson T, et al. Determinants of Spike infectivity, processing, and neutralization in SARS-CoV-2 Omicron subvariants BA.1 and BA.2. Cell Host Microbe. 2022;30:1255-1268.e5.

29. Dolton G, Rius C, Hasan MS, Wall A, Szomolay B, Behiry E, et al. Emergence of immune escape at dominant SARS-CoV-2 killer T cell epitope. Cell. 2022;185:2936-2951.e19.

30. Tye EXC, Jinks E, Haigh TA, Kaul B, Patel P, Parry HM, et al. Mutations in SARS-CoV-2 spike protein impair epitope-specific CD4+ T cell recognition. Nat Immunol. 2022;23:1726–34.

31. Pretti MAM, Galvani RG, Scherer NM, Farias AS, Boroni M. In silico analysis of mutant epitopes in new SARS-CoV-2 lineages suggest global enhanced CD8+ T cell reactivity and also signs of immune response escape. Infect Genet Evol. 2022;99:105236.

32. de Silva TI, Liu G, Lindsey BB, Dong D, Moore SC, Hsu NS, et al. The impact of viral mutations on recognition by SARS-CoV-2 specific T cells. iScience. 2021;24:103353.

33. Agerer B, Koblischke M, Gudipati V, Montaño-Gutierrez LF, Smyth M, Popa A, et al. SARS-CoV-2 mutations in MHC-I-restricted epitopes evade CD8+ T cell responses. Science Immunology. 2021;6:eabg6461.

34. Profiling CD8+ T cell epitopes of COVID-19 convalescents reveals reduced cellular immune responses to SARS-CoV-2 variants - PubMed. https://pubmed.ncbi.nlm.nih.gov/34506741/. Accessed 20 Jan 2023.

35. covSPECTRUM. https://cov-spectrum.org. Accessed 20 Jan 2023.

36. Kombe Kombe AJ, Biteghe FAN, Ndoutoume ZN, Jin T. CD8+ T-cell immune escape by SARS-CoV-2 variants of concern. Frontiers in Immunology. 2022;13.

37. Link-Gelles R. Effectiveness of Bivalent mRNA Vaccines in Preventing Symptomatic SARS-CoV-2 Infection — Increasing Community Access to Testing Program, United States, September–November 2022. MMWR Morb Mortal Wkly Rep. 2022;71.

38. Efficacy and Safety of the mRNA-1273 SARS-CoV-2 Vaccine | NEJM. https://www.nejm.org/doi/full/10.1056/nejmoa2035389. Accessed 7 Jan 2023.

39. Polack FP, Thomas SJ, Kitchin N, Absalon J, Gurtman A, Lockhart S, et al. Safety and Efficacy of the BNT162b2 mRNA Covid-19 Vaccine. New England Journal of Medicine. 2020;383:2603–15.

40. Tenforde MW. Early Estimates of Bivalent mRNA Vaccine Effectiveness in Preventing COVID-19–Associated Emergency Department or Urgent Care Encounters and Hospitalizations Among Immunocompetent Adults — VISION Network, Nine States, September–November 2022. MMWR Morb Mortal Wkly Rep. 2022;71.

41. Patalon T, Saciuk Y, Peretz A, Perez G, Lurie Y, Maor Y, et al. Waning effectiveness of the third dose of the BNT162b2 mRNA COVID-19 vaccine. Nat Commun. 2022;13:3203.

42. Levine-Tiefenbrun M, Yelin I, Alapi H, Katz R, Herzel E, Kuint J, et al. Viral loads of Delta-variant SARS-CoV-2 breakthrough infections after vaccination and booster with BNT162b2. Nat Med. 2021;27:2108–10.

43. Mizrahi B, Lotan R, Kalkstein N, Peretz A, Perez G, Ben-Tov A, et al. Correlation of SARS-CoV-2-breakthrough infections to time-from-vaccine. Nat Commun. 2021;12:6379.

44. Chemaitelly H, Tang P, Hasan MR, AlMukdad S, Yassine HM, Benslimane FM, et al. Waning of BNT162b2 Vaccine Protection against SARS-CoV-2 Infection in Qatar. N Engl J Med. 2021;385:e83.

45. Levin EG, Lustig Y, Cohen C, Fluss R, Indenbaum V, Amit S, et al. Waning Immune Humoral Response to BNT162b2 Covid-19 Vaccine over 6 Months. N Engl J Med. 2021;385:e84.

46. Bar-On YM, Goldberg Y, Mandel M, Bodenheimer O, Freedman L, Kalkstein N, et al. Protection of BNT162b2 Vaccine Booster against Covid-19 in Israel. N Engl J Med. 2021;385:1393–400.

47. Eurosurveillance | COVID-19 vaccine effectiveness against severe disease from SARS-CoV-2 Omicron BA.1 and BA.2 subvariants – surveillance results from southern Sweden, December 2021 to March 2022. https://www.eurosurveillance.org/content/10.2807/1560-7917.ES.2022.27.18.2200322. Accessed 7 Jan 2023.

48. COVID-19 vaccine surveillance report: week 15. UK Health Security Agency; 2022. Available: https://assets.publishing.service.gov.uk/government/uploads/system/uploads/attachment_data/file/1069256/Vaccine_surveillance_report_-_week_15.pdf

49. Wright BJ, Tideman S, Diaz GA, French T, Parsons GT, Robicsek A. Comparative vaccine effectiveness against severe COVID-19 over time in US hospital administrative data: a case-control study. Lancet Respir Med. 2022;10:557–65.

50. Suah JL, Husin M, Tok PSK, Tng BH, Thevananthan T, Low EV, et al. Waning COVID-19 Vaccine Effectiveness for BNT162b2 and CoronaVac in Malaysia: An Observational Study. Int J Infect Dis. 2022;119:69–76.

51. Cromer D, Steain M, Reynaldi A, Schlub TE, Wheatley AK, Juno JA, et al. Neutralising antibody titres as predictors of protection against SARS-CoV-2 variants and the impact of boosting: a meta-analysis. The Lancet Microbe. 2022;3:e52–61.

52. Feikin DR, Higdon MM, Abu-Raddad LJ, Andrews N, Araos R, Goldberg Y, et al. Duration of effectiveness of vaccines against SARS-CoV-2 infection and COVID-19 disease: results of a systematic review and meta-regression. The Lancet. 2022;399:924–44.

53. Tseng HF, Ackerson BK, Luo Y, Sy LS, Talarico CA, Tian Y, et al. Effectiveness of mRNA-1273 against SARS-CoV-2 Omicron and Delta variants. Nat Med. 2022;28:1063–71.

54. Lauring AS, Tenforde MW, Chappell JD, Gaglani M, Ginde AA, McNeal T, et al. Clinical severity of, and effectiveness of mRNA vaccines against, covid-19 from omicron, delta, and alpha SARS-CoV-2 variants in the United States: prospective observational study. BMJ. 2022;376:e069761.

55. Bacher P, Rosati E, Esser D, Martini GR, Saggau C, Schiminsky E, et al. Low-Avidity CD4+ T Cell Responses to SARS-CoV-2 in Unexposed Individuals and Humans with Severe COVID-19. Immunity. 2020;53:1258-1271.e5.

56. Grifoni A, Weiskopf D, Ramirez SI, Mateus J, Dan JM, Moderbacher CR, et al. Targets of T Cell Responses to SARS-CoV-2 Coronavirus in Humans with COVID-19 Disease and Unexposed Individuals. Cell. 2020;181:1489-1501.e15.

57. Murray SM, Ansari AM, Frater J, Klenerman P, Dunachie S, Barnes E, et al. The impact of pre-existing cross-reactive immunity on SARS-CoV-2 infection and vaccine responses. Nat Rev Immunol. 2022;:1–13.

58. Kundu R, Narean JS, Wang L, Fenn J, Pillay T, Fernandez ND, et al. Cross-reactive memory T cells associate with protection against SARS-CoV-2 infection in COVID-19 contacts. Nat Commun. 2022;13:80.

59. Nelde A, Bilich T, Heitmann JS, Maringer Y, Salih HR, Roerden M, et al. SARS-CoV-2-derived peptides define heterologous and COVID-19-induced T cell recognition. Nat Immunol. 2021;22:74–85.

60. Le Bert N, Tan AT, Kunasegaran K, Tham CYL, Hafezi M, Chia A, et al. SARS-CoV-2-specific T cell immunity in cases of COVID-19 and SARS, and uninfected controls. Nature. 2020;584:457–62.

61. Zahran AM, Nafady-Hego H, Rashad A, El-Badawy O, Nasif KA, Mostafa AT, et al. Increased percentage of apoptotic and CTLA-4 (CD152) expressing cells in CD4+/CD8+ cells in COVID-19 patients. Medicine. 2022;101:e30650.

62. Chen G, Wu D, Guo W, Cao Y, Huang D, Wang H, et al. Clinical and immunological features of severe and moderate coronavirus disease 2019. J Clin Invest. 130:2620–9.

63. Zheng M, Gao Y, Wang G, Song G, Liu S, Sun D, et al. Functional exhaustion of antiviral lymphocytes in COVID-19 patients. Cell Mol Immunol. 2020;17:533–5.

64. Wang F, Nie J, Wang H, Zhao Q, Xiong Y, Deng L, et al. Characteristics of Peripheral Lymphocyte Subset Alteration in COVID-19 Pneumonia. J Infect Dis. 2020;221:1762–9.

65. Arshad N, Laurent-Rolle M, Ahmed WS, Hsu JC-C, Mitchell SM, Pawlak J, et al. SARS-CoV-2 accessory proteins ORF7a and ORF3a use distinct mechanisms to down-regulate MHC-I surface expression. Proc Natl Acad Sci USA. 2023;120:e2208525120.

66. Zhang Y, Chen Y, Li Y, Huang F, Luo B, Yuan Y, et al. The ORF8 protein of SARS-CoV-2 mediates immune evasion through down-regulating MHC-I. Proceedings of the National Academy of Sciences. 2021;118:e2024202118.

67. Maher AK, Burnham KL, Jones EM, Tan MMH, Saputil RC, Baillon L, et al. Transcriptional reprogramming from innate immune functions to a pro-thrombotic signature by monocytes in COVID-19. Nat Commun. 2022;13:7947.

68. Lymphocytopenia - Hematology and Oncology. Merck Manuals Professional Edition. https://www.merckmanuals.com/professional/hematology-and-oncology/leukopenias/lymphocytopenia. Accessed 14 Jan 2023.

69. Shen X-R, Geng R, Li Q, Chen Y, Li S-F, Wang Q, et al. ACE2-independent infection of T lymphocytes by SARS-CoV-2. Signal Transduct Target Ther. 2022;7:83.

70. Brunetti NS, Davanzo GG, de Moraes D, Ferrari AJR, de Souza GF, Muraro SP, et al. SARS-CoV-2 Uses CD4 to Infect T Helper Lymphocytes. preprint. Infectious Diseases (except HIV/AIDS); 2020.

71. Diao B, Wang C, Tan Y, Chen X, Liu Y, Ning L, et al. Reduction and Functional Exhaustion of T Cells in Patients With Coronavirus Disease 2019 (COVID-19). Front Immunol. 2020;11:827.

72. Zheng H-Y, Zhang M, Yang C-X, Zhang N, Wang X-C, Yang X-P, et al. Elevated exhaustion levels and reduced functional diversity of T cells in peripheral blood may predict severe progression in COVID-19 patients. Cell Mol Immunol. 2020;17:541–3.

73. Cizmecioglu A, Akay Cizmecioglu H, Goktepe MH, Emsen A, Korkmaz C, Esenkaya Tasbent F, et al. Apoptosis-induced T-cell lymphopenia is related to COVID-19 severity. J Med Virol. 2021;93:2867–74.

74. André S, Picard M, Cezar R, Roux-Dalvai F, Alleaume-Butaux A, Soundaramourty C, et al. T cell apoptosis characterizes severe Covid-19 disease. Cell Death Differ. 2022;29:1486–99.

75. Tan L, Wang Q, Zhang D, Ding J, Huang Q, Tang Y-Q, et al. Lymphopenia predicts disease severity of COVID-19: a descriptive and predictive study. Signal Transduct Target Ther. 2020;5:33.

76. Files JK, Boppana S, Perez MD, Sarkar S, Lowman KE, Qin K, et al. Sustained cellular immune dysregulation in individuals recovering from SARS-CoV-2 infection. J Clin Invest. 2021;131.

77. Phetsouphanh C, Darley DR, Wilson DB, Howe A, Munier CML, Patel SK, et al. Immunological dysfunction persists for 8 months following initial mild-to-moderate SARS-CoV-2 infection. Nat Immunol. 2022;23:210–6.

78. Danwang C, Noubiap JJ, Robert A, Yombi JC. Outcomes of patients with HIV and COVID-19 co-infection: a systematic review and meta-analysis. AIDS Res Ther. 2022;19:3.

79. McMahan K, Yu J, Mercado NB, Loos C, Tostanoski LH, Chandrashekar A, et al. Correlates of protection against SARS-CoV-2 in rhesus macaques. Nature. 2021;590:630–4.

80. Hasenkrug KJ, Feldmann F, Myers L, Santiago ML, Guo K, Barrett BS, et al. Recovery from Acute SARS-CoV-2 Infection and Development of Anamnestic Immune Responses in T Cell-Depleted Rhesus Macaques. mBio. 2021;12:e0150321.

81. Reynolds J, Shojania K, Marra CA. Abatacept: A Novel Treatment for Moderate-to-Severe Rheumatoid Arthritis. Pharmacotherapy: The Journal of Human Pharmacology and Drug Therapy. 2007;27:1693–701.

82. Chitale S, Moots R. Abatacept: the first T lymphocyte co-stimulation modulator, for the treatment of rheumatoid arthritis. Expert Opinion on Biological Therapy. 2008;8:115–22.

83. Vincenti F. Costimulation blockade in autoimmunity and transplantation. Journal of Allergy and Clinical Immunology. 2008;121:299–306.

84. Sparks JA, Wallace ZS, Seet AM, Gianfrancesco MA, Izadi Z, Hyrich KL, et al. Associations of baseline use of biologic or targeted synthetic DMARDs with COVID-19 severity in rheumatoid arthritis: Results from the COVID-19 Global Rheumatology Alliance physician registry. Ann Rheum Dis. 2021;80:1137–46.

85. Ko ER, Anstrom KJ, Panettieri RA, Lachiewicz AM, Maillo M, O'Halloran JA, et al. Abatacept for Treatment of Adults Hospitalized with Moderate or Severe Covid-19. 2022;:2022.09.22.22280247.

86. Bristol Myers Squibb Announces Topline Results Showing Treatment with Orencia (abatacept) Improved Survival in People Hospitalized with COVID-19. https://news.bms.com/news/details/2022/Bristol-Myers-Squibb-Announces-Topline-Results-Showing-Treatment-with-Orencia-abatacept-Improved-Survival-in-People-Hospitalized-with-COVID-19/default.aspx. Accessed 7 Jan 2023.

87. Hamdy A, Leonardi A. Superantigens and SARS-CoV-2. Pathogens. 2022;11:390.

88. Schreibing F, Hannani MT, Kim H, Nagai JS, Ticconi F, Fewings E, et al. Dissecting CD8+ T cell pathology of severe SARS-CoV-2 infection by single-cell immunoprofiling. Front Immunol. 2022;13:1066176.

89. Georg P, Astaburuaga-García R, Bonaguro L, Brumhard S, Michalick L, Lippert LJ, et al. Complement activation induces excessive T cell cytotoxicity in severe COVID-19. Cell. 2022;185:493-512.e25.

90. Kalfaoglu B, Almeida-Santos J, Tye CA, Satou Y, Ono M. T-cell dysregulation in COVID-19. Biochemical and Biophysical Research Communications. 2021;538:204–10.

91. Dowell AC, Ireland G, Zuo J, Moss P, Ladhani S, sKIDs Investigation Team. Association of Spike-Specific T Cells With Relative Protection From Subsequent SARS-CoV-2 Omicron Infection in Young Children. JAMA Pediatrics. 2023;177:96–7.

92. Hajian-Tilaki K. Receiver Operating Characteristic (ROC) Curve Analysis for Medical Diagnostic Test Evaluation. Caspian J Intern Med. 2013;4:627–35.

93. Pardieck IN, van der Sluis TC, van der Gracht ETI, Veerkamp DMB, Behr FM, van Duikeren S, et al. A third vaccination with a single T cell epitope confers protection in a murine model of SARS-CoV-2 infection. Nat Commun. 2022;13:3966.

94. Bilich T, Roerden M, Maringer Y, Nelde A, Heitmann JS, Dubbelaar ML, et al. Preexisting and Post-COVID-19 Immune Responses to SARS-CoV-2 in Patients with Cancer. Cancer Discov. 2021;11:1982–95.

95. Bange EM, Han NA, Wileyto P, Kim JY, Gouma S, Robinson J, et al. CD8+ T cells contribute to survival in COVID-19 patients with hematologic cancers. Nat Med. 2021;27:1280–9.

96. Terpos E, Stellas D, Rosati M, Sergentanis TN, Hu X, Politou M, et al. SARS-CoV-2 antibody kinetics eight months from COVID-19 onset: Persistence of spike antibodies but loss of neutralizing antibodies in 24% of convalescent plasma donors. Eur J Intern Med. 2021;89:87–96.

97. Zeng C, Evans JP, Reisinger S, Woyach J, Liscynesky C, Boghdadly ZE, et al. Impaired neutralizing antibody response to COVID-19 mRNA vaccines in cancer patients. Cell & Bioscience. 2021;11:197.

98. Addetia A, Crawford KHD, Dingens A, Zhu H, Roychoudhury P, Huang M-L, et al. Neutralizing Antibodies Correlate with Protection from SARS-CoV-2 in Humans during a Fishery Vessel Outbreak with a High Attack Rate. J Clin Microbiol. 2020;58:e02107-20.

99. An immune correlate of SARS-CoV-2 infection and severity of reinfections | medRxiv. https://www.medrxiv.org/content/10.1101/2021.11.23.21266767v1.full. Accessed 7 Jan 2023.

100. Early Humoral Response Correlates with Disease Severity and Outcomes in COVID-19 Patients. https://www.ncbi.nlm.nih.gov/pmc/articles/PMC7761967/. Accessed 7 Jan 2023.

101. Khoury DS, Cromer D, Reynaldi A, Schlub TE, Wheatley AK, Juno JA, et al. Neutralizing antibody levels are highly predictive of immune protection from symptomatic SARS-CoV-2 infection. Nat Med. 2021;27:1205–11.

102. Gilbert PB, Montefiori DC, McDermott AB, Fong Y, Benkeser D, Deng W, et al. Immune correlates analysis of the mRNA-1273 COVID-19 vaccine efficacy clinical trial. Science. 2022;375:43–50.

103. Feng S, Phillips DJ, White T, Sayal H, Aley PK, Bibi S, et al. Correlates of protection against symptomatic and asymptomatic SARS-CoV-2 infection. Nat Med. 2021;27:2032–40.

104. Cohen JA, Stuart RM, Rosenfeld K, Lyons H, White M, Kerr CC, et al. Quantifying the role of naturally- and vaccine-derived neutralizing antibodies as a correlate of protection against COVID-19 variants. 2021;:2021.05.31.21258018.

105. Plotkin SA. Correlates of Protection Induced by Vaccination. Clin Vaccine Immunol. 2010;17:1055–65.

106. Cele S, Jackson L, Khoury DS, Khan K, Moyo-Gwete T, Tegally H, et al. Omicron extensively but incompletely escapes Pfizer BNT162b2 neutralization. Nature. 2022;602:654–6.

107. Cromer D, Reynaldi A, Steain M, Triccas JA, Davenport MP, Khoury DS. Relating In Vitro Neutralization Level and Protection in the CVnCoV (CUREVAC) Trial. Clin Infect Dis. 2022;75:e878–9.

108. Koutsakos M, Lee WS, Reynaldi A, Tan H-X, Gare G, Kinsella P, et al. The magnitude and timing of recalled immunity after breakthrough infection is shaped by SARS-CoV-2 variants. Immunity. 2022;55:1316-1326.e4.

109. Bruel T, Pinaud L, Tondeur L, Planas D, Staropoli I, Porrot F, et al. Neutralising antibody responses to SARS-CoV-2 omicron among elderly nursing home residents following a booster dose of BNT162b2 vaccine: A community-based, prospective, longitudinal cohort study. eClinicalMedicine. 2022;51.

110. Bitoun S, Henry J, Desjardins D, Vauloup-Fellous C, Dib N, Belkhir R, et al. Rituximab Impairs B Cell Response But Not T Cell Response to COVID-19 Vaccine in Autoimmune Diseases. Arthritis Rheumatol. 2022;74:927–33.

111. Andersen KM, Bates BA, Rashidi ES, Olex AL, Mannon RB, Patel RC, et al. Long-term use of immunosuppressive medicines and in-hospital COVID-19 outcomes: a retrospective cohort study using data from the National COVID Cohort Collaborative. The Lancet Rheumatology. 2022;4:e33–41.

112. Ekin A, Coskun BN, Dalkilic E, Pehlivan Y. The effects of COVID-19 infection on the mortality of patients receiving rituximab therapy. Ir J Med Sci. 2022;:1–15.

113. Strangfeld A, Schäfer M, Gianfrancesco MA, Lawson-Tovey S, Liew JW, Ljung L, et al. Factors associated with COVID-19-related death in people with rheumatic diseases: results from the COVID-19 Global Rheumatology Alliance physician-reported registry. Ann Rheum Dis. 2021;80:930–42.

114. Avouac J, Drumez E, Hachulla E, Seror R, Georgin-Lavialle S, El Mahou S, et al. COVID-19 outcomes in patients with inflammatory rheumatic and musculoskeletal diseases treated with rituximab: a cohort study. Lancet Rheumatol. 2021;3:e419–26.

115. Sormani MP, De Rossi N, Schiavetti I, Carmisciano L, Cordioli C, Moiola L, et al. Disease-Modifying Therapies and Coronavirus Disease 2019 Severity in Multiple Sclerosis. Annals of Neurology. 2021;89:780–9.

116. Bsteh G, Dürauer S, Assar H, Hegen H, Heschl B, Leutmezer F, et al. Humoral immune response after COVID-19 in multiple sclerosis: A nation-wide Austrian study. Mult Scler. 2021;27:2209–18.

117. Gadani SP, Reyes-Mantilla M, Jank L, Harris S, Douglas M, Smith MD, et al. Discordant humoral and T cell immune responses to SARS-CoV-2 vaccination in people with multiple sclerosis on anti-CD20 therapy. EBioMedicine. 2021;73:103636.

118. Hada M, Mosholder AD, Leishear K, Perez-Vilar S. Systematic review of risk of SARS-CoV-2 infection and severity of COVID-19 with therapies approved to treat multiple sclerosis. Neurol Sci. 2022;43:1557–67.

119. Mohanraj D, Baldwin S, Singh S, Gordon A, Whitelegg A. Cellular and humoral responses to SARS-CoV-2 vaccination in immunosuppressed patients. Cell Immunol. 2022;373:104501.

120. Calabrese CM, Kirchner E, Husni EM, Moss BP, Fernandez AP, Jin Y, et al. Breakthrough SARS-CoV-2 Infections in Patients With Immune-Mediated Disease Undergoing B Cell-Depleting Therapy: A Retrospective Cohort Analysis. Arthritis Rheumatol. 2022;74:1906–15.

121. Di Fusco M, Lin J, Vaghela S, Lingohr-Smith M, Nguyen JL, Scassellati Sforzolini T, et al. COVID-19 vaccine effectiveness among immunocompromised populations: a targeted literature review of real-world studies. Expert Rev Vaccines. :1–17.

122. Mues KE, Kirk B, Patel DA, Gelman A, Chavers LS, Talarico CA, et al. Real-world comparative effectiveness of mRNA-1273 and BNT162b2 vaccines among immunocompromised adults identified in administrative claims data in the United States. Vaccine. 2022;40:6730–9.

123. Hoff LS, Ravichandran N, Shinjo SK, Day J, Sen P, Junior JG, et al. COVID-19 severity and vaccine breakthrough infections in idiopathic inflammatory myopathies, other systemic autoimmune and inflammatory diseases, and healthy controls: a multicenter cross-sectional study from the COVID-19 Vaccination in Autoimmune Diseases (COVAD) survey. Rheumatol Int. 2022;:1–12.

124. Galmiche S, Luong Nguyen LB, Tartour E, de Lamballerie X, Wittkop L, Loubet P, et al. Immunological and clinical efficacy of COVID-19 vaccines in immunocompromised populations: a systematic review. Clin Microbiol Infect. 2022;28:163–77.

125. Focosi D, McConnell S, Casadevall A, Cappello E, Valdiserra G, Tuccori M. Monoclonal antibody therapies against SARS-CoV-2. Lancet Infect Dis. 2022;22:e311–26.

126. Dagotto G, Ventura JD, Martinez DR, Anioke T, Chung BS, Siamatu M, et al. Immunogenicity and protective efficacy of a rhesus adenoviral vaccine targeting conserved COVID-19 replication transcription complex. npj Vaccines. 2022;7:1–9.

127. Thieme CJ, Anft M, Paniskaki K, Blazquez-Navarro A, Doevelaar A, Seibert FS, et al. Robust T Cell Response Toward Spike, Membrane, and Nucleocapsid SARS-CoV-2 Proteins Is Not Associated with Recovery in Critical COVID-19 Patients. Cell Rep Med. 2020;1:100092.

128. Govender M, Hopkins FR, Göransson R, Svanberg C, Shankar EM, Hjorth M, et al. T cell perturbations persist for at least 6 months following hospitalization for COVID-19. Frontiers in Immunology. 2022;13.

129. Helfand M, Fiordalisi C, Wiedrick J, Ramsey KL, Armstrong C, Gean E, et al. Risk for Reinfection After SARS-CoV-2: A Living, Rapid Review for American College of Physicians Practice Points on the Role of the Antibody Response in Conferring Immunity Following SARS-CoV-2 Infection. Ann Intern Med. 2022;175:547–55.

130. Flacco ME, Acuti Martellucci C, Baccolini V, De Vito C, Renzi E, Villari P, et al. Risk of reinfection and disease after SARS-CoV-2 primary infection: Meta-analysis. European Journal of Clinical Investigation. 2022;52:e13845.
